# Root proteomic and metabolic analyses reveal specific responses to drought stress in differently tolerant grapevine rootstocks

**DOI:** 10.1186/s12870-018-1343-0

**Published:** 2018-06-20

**Authors:** Bhakti Prinsi, Alfredo Simone Negri, Osvaldo Failla, Attilio Scienza, Luca Espen

**Affiliations:** Dipartimento di Scienze Agrarie e Ambientali - Produzione, Territorio, Agroenergia (DiSAA), Università degli Studi di Milano, Via Celoria, 2, 20133 Milano, Italy

**Keywords:** Drought, Grapevine, Proteomics, Rootstock, Water stress

## Abstract

**Background:**

Roots play a central role in plant response to water stress (WS). They are involved in its perception and signalling to the leaf as well as in allowing the plant to adapt to maintaining an adequate water balance. Only a few studies have investigated the molecular/biochemical responses to WS in roots of perennial plants, such as grapevine. This study compares two grapevine rootstock genotypes (i.e. 101.14 and M4) with different tolerance to WS, evaluating the responses at proteomic and metabolite levels.

**Results:**

WS induced changes in the abundance of several proteins in both genotypes (17 and 22% of the detected proteins in 101.14 and M4, respectively). The proteomic analysis revealed changes in many metabolic pathways that fitted well with the metabolite data. M4 showed metabolic responses which were potentially able to counteract the WS effects, such as the drop in cell turgor, increased oxidative stress and loss of cell structure integrity/functionality. However, in 101.14 it was evident that the roots were suffering more severely from these effects. We found that many proteins classified as active in energy metabolism, hormone metabolism, protein, secondary metabolism and stress functional classes showed particular differences between the two rootstocks.

**Conclusion:**

The proteomic/metabolite comparative analysis carried out provides new information on the possible biochemical and molecular strategies adopted by grapevine roots to counteract WS. Although further work is needed to define in detail the role(s) of the proteins and metabolites that characterize WS response, this study, involving the M4 rootstock genotype, highlights that osmotic responses, modulations of C metabolism, mitochondrial functionality and some specific responses to stress occurring in the roots play a primary role in *Vitis* spp. tolerance to this type of abiotic stress.

**Electronic supplementary material:**

The online version of this article (10.1186/s12870-018-1343-0) contains supplementary material, which is available to authorized users.

## Background

Among the environmental problems related to viticulture, drought is one of the major factors that negatively affect grape production [1]. However, a significant part of the land devoted to viticulture, such as the Mediterranean regions of Europe, is located in areas characterized by a seasonal drought coinciding with the grapevine ripening period, thus affecting both yield and fruit quality [2–5]. Moreover, provisional studies on climate changes indicate that in these areas the availability of water will diminish in the coming years [4, 6].

Under drought, the grapevine leaf exhibits deep changes at molecular, biochemical, physiological and morphological levels which are useful to improve water use efficiency (WUE) through the activation of adaptive responses [2, 7–13]. Furthermore, it is clear that the strategy adopted in these adverse conditions can be somewhat different between grapevine cultivars (isohydric or anisohydric behaviours), besides being affected by pedo-climatic conditions [3, 9]. Among the typical immediate responses observed in the leaf organ under water deficit, there is progressive stomatal closure to counteract the untenable water loss [2, 7, 10]. This response, mediated by both hydraulic and chemical signalling, has direct and severe effects on photosynthesis and therefore on biomass production [2, 12, 14, 15]. Moreover, the reduction in water content leads to a loss of turgor with a consequent reduction of plant growth [7, 16]. In this condition, other typical responses in leaf tissues are osmotic adjustment, activation of ROS-scavenging mechanisms, changes in cell wall elasticity and metabolic acclimation [7, 9, 17, 18].

The recent literature reinforces the central role in the WS response of the roots, which take part in stress perception, adaptation and signaling to the aerial system, as well as their role in the uptake and transport of water towards the leaves [9, 14, 19–21]. In this context, many studies have highlighted that a positive plant response to WS is strictly dependent on the roots’ capabilities of sustaining growth (i.e.*,* changing the depth and density of the root system) and of maintaining/increasing the root hydraulic conductance [10, 13, 19–23]. Regarding this last aspect, the woody roots of perennial plants like grapevine, despite their low hydraulic conductance, can significantly contribute to water absorption from the soil [24].

Many studies indicate that the tolerance to WS of the root depends on its capability of maintaining adequate symplastic osmotic potential, cell wall protein composition, carbohydrate metabolism and the metabolic pathways involved in the oxidative stress response [25–28]. In agreement with this picture, two recent studies have also shown that in grapevine roots these responses play a central role in WS tolerance [29, 30].

In recent years, proteomic approaches have been undertaken to study WS responses. Some of this work focused attention on the roots of herbaceous species, such as soybean, wild watermelon, wheat, rapeseed and sugarcane [31]. These studies reported proteome changes linked to different biochemical responses, such as an increase in energy demand, transport activities, and the appearance or increase in the levels of proteins known to have protective roles under these stress conditions. In grapevine, the proteomic approach has been used to investigate the effects of WS in shoot and fruit tissues [18, 32, 33]. To our knowledge, to date no proteomic investigation has studied water stress responses in the roots of this perennial species.

Previously, the molecular, biochemical and physiological responses to WS of the novel rootstock genotype named M4 [(*V. vinifera* x *V. berlandieri*) x *V. berlandieri* cv. Resseguier no. 1] have been evaluated [29, 30]. The comparison with the genotype 101.14 (*V. riparia x V. rupestris*) highlighted the greater tolerance to WS of M4, as shown by photosynthetic parameters as well as by the analysis of molecular and biochemical responses in both leaf and root [29, 30].

The present study focused attention on the changes in root proteomes which occur in M4 and 101.14 rootstock genotypes under WS. The analyses were performed on samples obtained from the same experiments described by Meggio et al. and Corso et al. [29, 30], so allowing a high level of confidence between the proteomic results and the physiological, biochemical and molecular ones. The analyses were carried out to evaluate the proteomic changes induced by harsh WS conditions (i.e. reduction of the field soil capacity down to 30%). Moreover, a metabolomic analysis was performed to obtain further information on the effects induced by WS on the main biochemical pathways, such as glycolysis and related sugar metabolism, the Krebs cycle and amino acid metabolisms. The results showed many differences between the two genotypes, so revealing specific traits linked to a low (101.14) or a high (M4) capability to tolerate WS conditions.

## Results

Mass spectrum interpretation allowed us to identify and quantify a total of 972 and 788 unique proteins for the 101.14 and the M4 genotypes, respectively. The technical parameters concerning peptide validation and protein identification reported in (Additional file 1: Table S1) indicated the good reliability of the approach adopted.

The results obtained by the comparison within the two experimental conditions experienced by each genotype (i.e., control versus water-stressed plants) are reported in Tables 1 and 2 and (Additional file 2: Table S2 A and B).

**Table 1:** Proteins showing significant changes in responses to WS in the 101.14 genotype

n.	Name (f.c.)	Accession	Δ:WS/C
*Carbon and energy metabolism* (1, 2, 3, 4, 5, 6, 7, 8, 9, 25)			
97	unnamed protein product - sucrose synthase 2-like (2)	CBI35298.3	new
397	fumarate hydratase 1, mitochondrial (8)	XP_002273033.1	new
595	alpha-1,4 glucan phosphorylase L isozyme, chloroplastic/amyloplastic-like (2)	XP_002279075.2	39.40
559	aldehyde dehydrogenase family 7 member A1 (5, 8, 20)	XP_002278093.1	6.14
938	ATP-citrate synthase alpha chain protein 2 isoform 2 (8)	XP_003633614.1	5.55
222	glucose-6-phosphate 1-dehydrogenase, cytoplasmic isoform (7)	XP_002266527.1	3.56
368	sucrose synthase 2 (2)	XP_002271896.1	2.86
818	1,4-alpha-glucan-branching enzyme-like (2)	XP_002284841.2	2.43
443	triosephosphate isomerase, chloroplastic-like isoform 1 (1)	XP_002274871.1	−2.12
432	enolase 1, chloroplastic-like (4)	XP_002274334.1	−2.18
269	fructokinase-2 (2)	XP_002268097.1	−2.38
158	pyruvate dehydrogenase E1 component subunit beta, mitochondrial-like isoform 1 (8)	XP_002264210.1	−2.53
350	dihydrolipoyllysine-residue acetyltransferase component of pyruvate dehydrogenase complex, mitochondrial-like isoform 1 (8)	XP_002271286.1	−2.94
698	dihydrolipoyllysine-residue acetyltransferase component of pyruvate dehydrogenase complex, mitochondrial-like (8, 11)	XP_002282287.1	−3.24
307	pyrophosphate--fructose 6-phosphate 1-phosphotransferase subunit beta-like (4)	XP_002269934.2	−3.40
622	pyruvate kinase isozyme A, chloroplastic isoform 1 (4, 11)	XP_002279975.1	−3.71
507	dihydrolipoyl dehydrogenase (8, 11, 21)	XP_002276853.1	−4.56
301	pyruvate dehydrogenase E1 component subunit beta (1, 8, 11)	XP_002269441.1	−8.46
236	glucose-6-phosphate 1-dehydrogenase, chloroplastic (7, 30)	XP_002266930.1	−25.91
280	succinate dehydrogenase [ubiquinone] iron-sulfur subunit 1, mitochondrial (8, 29)	XP_002268523.1	d.
551	carbonic anhydrase, chloroplastic (8, 16)	XP_002277957.1	d.
*Cell Wall* (10)			
273	UDP-glucose 4-epimerase GEPI48 (10)	XP_002268294.2	4.34
282	beta-xylosidase/alpha-L-arabinofuranosidase 2-like (10, 33)	XP_002268626.2	−2.68
620	probable UDP-arabinopyranose mutase 5 (10)	XP_002279911.1	−2.27
423	probable xyloglucan endotransglucosylase/hydrolase protein 30-like (10)	XP_002273975.1	−3.28
702	probable rhamnose biosynthetic enzyme 1 (10)	XP_002282339.1	−5.93
*Lipid Metabolism* (11)			
248	long chain acyl-CoA synthetase 8 (11)	XP_002267417.1	new
312	glyoxysomal fatty acid beta-oxidation multifunctional protein MFP-a (11)	XP_002270067.1	50.99
271	phospholipase D alpha 1 (11, 27)	XP_002268195.1	2.46
564	biotin carboxyl carrier protein of acetyl-CoA carboxylase-like (11)	XP_002278151.2	−2.86
183	3-oxoacyl-[acyl-carrier-protein] synthase I, chloroplastic-like (11)	XP_002265207.1	−3.09
942	phospholipase C 4-like isoform 2 (11)	XP_003633883.1	−3.12
219	biotin carboxylase 1, chloroplastic-like (11)	XP_002266489.1	−3.58
268	3-oxoacyl-[acyl-carrier-protein] reductase, chloroplastic (11, 26)	XP_002268080.1	−3.71
775	acyl-CoA-binding domain-containing protein 4-like isoform 1 (11)	XP_002284019.1	−4.32
958	flavoprotein wrbA isoform 2 (11)	XP_003634692.1	d.
*N and amino acid metabolism* (12, 13)			
3	S-adenosylmethionine synthase 2 (13, 15)	A7NVX9.1	−2.13
929	glyoxylate reductase isoform 2 (1, 13, 26)	XP_003632860.1	−2.34
971	phosphoserine aminotransferase, chloroplastic-like (13, 27)	XP_003635669.1	−2.36
843	ferredoxin--nitrite reductase, chloroplastic (12)	XP_002285208.1	−2.65
518	serine hydroxymethyltransferase, mitochondrial (1, 13, 25)	XP_002277146.1	−2.92
318	bifunctional 3-dehydroquinate dehydratase/shikimate dehydrogenase, chloroplastic (13)	XP_002270188.1	−4.84
206	adenosylhomocysteinase isoform 1 (13)	XP_002266154.1	−5.43
321	bifunctional 3-dehydroquinate dehydratase/shikimate dehydrogenase, chloroplastic-like (13)	XP_002270232.1	−6.15
212	probable S-adenosylmethionine-dependent methyltransferase At5g37990-like (13, 17)	XP_002266288.2	−7.51
342	methionine S-methyltransferase-like (13)	XP_002270977.1	−10.04
687	ornithine carbamoyltransferase, chloroplastic (13)	XP_002281919.1	−10.33
186	alanine aminotransferase 2 (13)	XP_002265294.2	−10.94
*Secondary metabolism* (16)			
576	2-C-methyl-D-erythritol 2,4-cyclodiphosphate synthase, chloroplastic-like (16)	XP_002278406.1	new
392	aldo-keto reductase family 4 member C9-like (3, 16)	XP_002272909.1	−2.10
136	3-isopropylmalate dehydratase small subunit (16)	XP_002263405.1	−2.26
549	isopentenyl-diphosphate Delta-isomerase I-like (16)	XP_002277935.1	−2.59
717	1-deoxy-D-xylulose 5-phosphate reductoisomerase, chloroplastic (16)	XP_002282761.1	−2.76
836	4-hydroxy-3-methylbut-2-en-1-yl diphosphate synthase (16)	XP_002285130.1	−3.06
960	bifunctional dihydroflavonol 4-reductase/flavanone 4-reductase-like (16, 26, 29)	XP_003634871.1	−6.69
106	chalcone--flavonone isomerase 1 (16, 27)	P51117.1	d.
373	protein SRG1 (16)	XP_002272119.1	d.
525	zeta-carotene desaturase, chloroplastic/chromoplastic (16)	XP_002277348.2	d.
557	REF/SRPP-like protein At1g67360-like (16)	XP_002278036.1	d.
584	carotenoid 9,10(9′,10′)-cleavage dioxygenase 1-like (16, 17)	XP_002278628.1	d.
*Hormone metabolism* (17)			
617	auxin-repressed 12.5 kDa protein isoform 1 (17, 27, 33)	XP_002279836.1	new
591	auxin-induced protein PCNT115 isoform 1 (17, 26)	XP_002278850.1	2.93
148	linoleate 13S-lipoxygenase 2–1, chloroplastic (17)	XP_002263854.1	2.55
457	HVA22-like protein a (17)	XP_002275428.1	2.29
828	gibberellin 20 oxidase 3-like (16, 17, 26)	XP_002284983.1	−2.15
767	probable indole-3-acetic acid-amido synthetase GH3.1 (17)	XP_002283886.1	−2.23
825	gibberellin 20 oxidase 3 (16, 17, 26)	XP_002284968.1	−7.11
54	hypothetical protein VITISV_007808 (gibberellin 3-beta-dioxygenase 1) (17, 26)	CAN66061.1	−14.90
897	1-aminocyclopropane-1-carboxylate oxidase 5 (17)	XP_002285881.1	d.
*Stress* (20)			
132	22.0 kDa heat shock protein (20)	XP_002263376.1	new
436	auxin-binding protein ABP19a-like (20)	XP_002274457.1	new
513	putative germin-like protein 2–1 (12, 20, 27, 30, 34)	XP_002277055.1	new
636	18.2 kDa class I heat shock protein (20, 29)	XP_002280353.1	new
900	MLP-like protein 28-like (20)	XP_003631204.1	new
652	18.2 kDa class I heat shock protein (20, 29)	XP_002280821.1	21.56
810	MLP-like protein 34 (20)	XP_002284578.1	2.92
349	universal stress protein A-like protein (20, 27, 33)	XP_002271154.1	2.14
152	stress-related protein-like (16)	XP_002263944.1	2.12
798	germin-like protein subfamily 1 member 17 (12, 20, 27, 34)	XP_002284436.1	−2.50
677	chitinase 2 (20, 21)	XP_002281729.1	−3.28
566	germin-like protein 9–3 (15, 20)	XP_002278170.1	−4.10
44	hypothetical protein VITISV_005677 (germin-like protein 9–3) (15, 20)	CAN61171.1	−14.62
64	hypothetical protein VITISV_005471 (germin-like protein 1) (12, 20, 27, 34)	CAN71140.1	d.
237	putative germin-like protein 2–1 (12, 20, 27, 30, 34)	XP_002266984.1	d.
240	putative germin-like protein 2–1 (12, 20, 27, 30, 34)	XP_002267172.1	d.
867	pathogen-related protein (20)	XP_002285489.1	d.
*Redox* (21)			
205	glutathione S-transferase DHAR3, chloroplastic (21)	XP_002266106.1	new
482	glutaredoxin (21)	XP_002276266.1	3.29
754	peroxiredoxin-2E, chloroplastic (21)	XP_002283652.1	−2.01
334	catalase isozyme 1 isoform 1 (21)	XP_002270703.2	−3.41
*Miscellaneous enzyme families* (26)			
845	probable glutathione S-transferase (26, 28, 33)	XP_002285214.1	new
36	hypothetical protein VITISV_041925 - carboxymethylenebutenolidase (26)	CAN60148.1	2.41
633	peroxidase 3 (20, 26)	XP_002280274.1	2.27
23	glutathione S-transferase 5 (11, 16, 26)	ABW34390.1	2.11
445	(+)-neomenthol dehydrogenase-like isoform 2 (26)	XP_002274970.2	−2.01
396	minor allergen Alt a 7-like (11, 26, 27)	XP_002273030.1	−2.16
427	probable inactive purple acid phosphatase 1-like (26, 27)	XP_002274118.2	−2.29
338	epoxide hydrolase 2 (26)	XP_002270883.2	−2.29
286	probable glutathione S-transferase (26)	XP_002268911.1	−2.45
840	NADP-dependent alkenal double bond reductase P1 (26, 34)	XP_002285167.1	−47.43
*DNA/RNA* (27, 28)			
281	proactivator polypeptide-like 1 isoform 1 (28)	XP_002268581.1	new
623	ribonuclease 3 (27)	XP_002280078.1	new
755	putative DNA repair protein RAD23–3 isoform 1 (28, 29)	XP_002283656.1	−2.49
164	KH domain-containing protein At4g18375 isoform 1 (27)	XP_002264417.1	−5.48
*Protein* (29)			
99	unnamed protein product (pseudouridine synthase) (29)	CBI39540.3	new
759	outer envelope pore protein 16, chloroplastic (29)	XP_002283749.1	new
803	eukaryotic translation initiation factor 3 subunit E (29)	XP_002284533.1	new
829	vesicle-fusing ATPase-like (29)	XP_002284987.1	new
252	protease Do-like 1, chloroplastic-like (29)	XP_002267510.2	7.32
154	aspartic proteinase nepenthesin-1 (27, 29)	XP_002263964.1	4.86
924	40S ribosomal protein S15a-like isoform 2 (29)	XP_003632608.1	4.08
794	N-carbamoyl-L-amino acid hydrolase-like (29)	XP_002284376.1	3.95
196	26S proteasome non-ATPase regulatory subunit 1-like (29)	XP_002265758.2	2.39
279	serine carboxypeptidase-like 18 (29)	XP_002268517.1	−2.00
774	acylamino-acid-releasing enzyme-like isoform 1 (29)	XP_002284013.2	−2.25
768	aspartic proteinase nepenthesin-1-like (27, 29)	XP_002283889.2	−2.31
641	uncharacterized protein LOC100259133 (m.: 29, 33)	XP_002280454.1	−2.58
50	hypothetical protein VITISV_017087 (serine carboxypeptidase II-3-like) (29)	CAN63486.1	−2.86
651	pyrrolidone-carboxylate peptidase isoform 4 (29)	XP_002280794.1	−2.92
722	protein transport protein Sec24-like At3g07100-like (29)	XP_002282857.1	−3.18
701	serine carboxypeptidase II-3-like (29)	XP_002282331.1	−3.30
65	hypothetical protein VITISV_003230 (m.: 29*)*	CAN71580.1	−3.78
679	cucumisin-like (29)	XP_002281790.2	−4.22
522	cucumisin-like (29)	XP_002277242.2	−5.96
689	serine carboxypeptidase II-3-like (29)	XP_002281988.1	−6.70
311	probable serine/threonine-protein kinase At5g41260 (29)	XP_002270065.1	−12.57
720	serine carboxypeptidase-like 45-like (29)	XP_002282852.1	−15.26
58	hypothetical protein VITISV_026357 (m.: 29, 30, 33)	CAN68006.1	−51.68
470	cucumisin-like (29)	XP_002275807.1	−106.41
303	uncharacterized protein LOC100254416 (pathogenesis-related protein 17) (29)	XP_002269470.1	d.
721	subtilisin-like protease-like (29, 30)	XP_002282856.1	d.
*Cell / signaling / development* (30, 31, 33)			
140	70 kDa peptidyl-prolyl isomerase (29, 31)	XP_002263566.2	new
357	actin-depolymerizing factor 10 (31)	XP_002271495.1	new
744	transmembrane emp24 domain-containing protein A (31)	XP_002283487.1	new
163	uncharacterized protein LOC100255239 (calcium ion binding protein) (30)	XP_002264359.1	5.11
799	glutelin type-A 1 (28, 33)	XP_002284459.1	4.50
415	calnexin homolog 1 (30)	XP_002273708.1	4.47
644	uncharacterized protein LOC100266227 (Late embryogenesis abundant protein Lea14-A) (33)	XP_002280489.1	4.09
934	VAMP-like protein YKT61-like (31)	XP_003633163.1	3.26
740	oxysterol-binding protein-related protein 3C (31)	XP_002283434.1	−2.07
676	uncharacterized protein HI_0488 (phosphatase YqaB) (33)	XP_002281714.1	−2.18
332	coatomer subunit epsilon-1 isoform 1 (31)	XP_002270662.1	−2.35
599	nitrogen regulatory protein P-II homolog (30)	XP_002279289.1	−2.60
907	uncharacterized protein LOC100854676 (m.: 30)	XP_003631533.1	−3.20
735	DAG protein, chloroplastic isoform 1 (33)	XP_002283211.1	−3.83
245	tubulin beta-1 chain isoform 1 (31)	XP_002267304.1	−4.10
374	golgin candidate 6-like (29, 31)	XP_002272168.1	−4.22
560	PRA1 family protein B4-like (30, 31)	XP_002278095.1	d.
*Transport* (34)			
704	pyrophosphate-energized vacuolar membrane proton pump 1 (34)	XP_002282358.1	2.76
870	uncharacterized protein LOC100240897 (m.: 34)	XP_002285517.1	2.29
548	aquaporin TIP2–3 (34)	XP_002277904.2	−5.27
*Others* (15, 18, 23, 24)			
86	unnamed protein product (DJ-1 family protein) (18)	CBI20205.3	new
516	ferritin-3, chloroplastic (15)	XP_002277114.1	6.49
731	NAD-dependent dihydropyrimidine dehydrogenase subunit PreA (23)	XP_002283095.1	5.41
700	ectonucleotide pyrophosphatase/phosphodiesterase family member 3 (23)	XP_002282308.1	4.52
95	unnamed protein product (nucleoside diphosphate kinase) (23)	CBI34488.3	3.07
639	6,7-dimethyl-8-ribityllumazine synthase, chloroplastic-like (18)	XP_002280427.1	2.58
492	guanine deaminase (23)	XP_002276494.1	−2.41
123	nucleoside diphosphate kinase 2, chloroplastic isoform 1 (23)	XP_002263177.1	−3.31
894	biotin--protein ligase (18)	XP_002285834.1	−4.28
535	probable carboxylesterase 15 (24)	XP_002277507.1	−7.50
90	unnamed protein product (soluble inorganic pyrophosphatase) (23)	CBI25065.3	d.
161	selT-like protein (15)	XP_002264265.1	d.
*Hypothetical / Unknown function* (35)			
76	unknown protein (35)	CAQ58595.1	new
85	unnamed protein product (Protein tolB) (35)	CBI18981.3	new
230	protein LURP-one-related 15 (35)	XP_002266795.1	new
902	probable nucleoredoxin 1-like (35)	XP_003631263.1	2.99
127	uncharacterized protein At5g48480 (35)	XP_002263284.1	2.81
387	pre-mRNA-processing factor 39-like (35)	XP_002272685.1	−2.36
420	uncharacterized protein LOC100242710 (35)	XP_002273917.1	−2.44
361	putative phosphatidylglycerol/phosphatidylinositol transfer protein DDB_G0282179 isoform 1 (35)	XP_002271535.1	−3.07
841	putative clathrin assembly protein At2g25430-like (35)	XP_002285168.1	−3.35
199	uncharacterized protein LOC100265424 (35)	XP_002265851.1	−4.35
232	uncharacterized protein LOC100253185 (35)	XP_002266892.1	−4.68
412	S-norcoclaurine synthase (35)	XP_002273566.1	−4.92
48	hypothetical protein VITISV_010154 (35)	CAN62850.1	−6.76
553	transmembrane protein 111 (35)	XP_002277989.1	−7.13
184	NADPH:quinone oxidoreductase (35)	XP_002265225.1	−16.72
82	unnamed protein product (metal ion binding protein, putative) (35)	CBI17463.3	d.
582	clavaminate synthase-like protein At3g21360 (35)	XP_002278552.1	d.
890	uncharacterized protein LOC100254028 (35)	XP_002285734.1	d.

Numbers reported in brackets refer to bin code (i.e. major functional categories). n.: identification number. f.c.: bin code of functional categories. Name: for proteins without a name in brackets are indicated the results from BLAST alignment against NCBI *Viridiplantae* database; m.: classification obtained through grape/Arabidopsis or grape/potato matching by BLASTp algorithm (E.value < 10^–^20). Δ: fold changes in WS plants with respect to the Control ones (up: %(SI)WS/%(SI)C, down: - %(SI)C/%(SI)WS). new: not present in C; d.: disappeared, not present in WS

**Table 2:** Proteins showing significant changes in responses to WS in the M4 genotype

n.	Name (f.c.)	Accession	Δ:WS/C
*Carbon and energy metabolism* (1, 2, 3, 4, 5, 6, 7, 8, 9, 25)			
474	alpha-1,4 glucan phosphorylase L isozyme, chloroplastic/amyloplastic-like (2)	XP_002279075.2	new
542	isocitrate dehydrogenase [NAD] catalytic subunit 5, mitochondrial-like (8)	XP_002281826.1	14.46
231	succinate-semialdehyde dehydrogenase (acetylating)-like (5, 26)	XP_002268625.1	7.33
481	2-oxoglutarate dehydrogenase, mitochondrial-like (8)	XP_002279332.2	6.00
757	ATP-citrate synthase alpha chain protein 2 isoform 2 (8)	XP_003633614.1	4.05
463	formate dehydrogenase, mitochondrial (25)	XP_002278444.1	3.05
695	phosphoenolpyruvate carboxylase, housekeeping isozyme isoform 1 (4)	XP_002285441.1	2.84
446	aldehyde dehydrogenase family 7 member A1 (5, 8, 20)	XP_002278093.1	2.34
251	L-idonate 5-dehydrogenase (3, 5, 26)	XP_002269895.1	2.23
626	citrate synthase, glyoxysomal (6)	XP_002284064.1	2.20
556	UTP--glucose-1-phosphate uridylyltransferase isoform 1(4)	XP_002282276.1	2.20
187	glucose-6-phosphate 1-dehydrogenase, cytoplasmic isoform (7)	XP_002266527.1	2.16
655	phosphoglucomutase, cytoplasmic (4)	XP_002284729.1	2.01
374	pyruvate decarboxylase isozyme 1 (5)	XP_002275486.1	−2.01
718	glucose-6-phosphate isomerase isoform 1 (4)	XP_002285696.1	−2.12
313	pyruvate decarboxylase isozyme 2 (5)	XP_002272615.1	−2.33
116	ferredoxin--NADP reductase, root isozyme, chloroplastic-like (7)	XP_002263658.2	−2.40
354	enolase 1, chloroplastic-like (4)	XP_002274334.1	−2.63
269	D-threo-aldose 1-dehydrogenase (2)	XP_002270562.1	−2.65
92	L-idonate 5-dehydrogenase (3, 5)	Q1PSI9.2	−2.93
413	dihydrolipoyl dehydrogenase (8, 11, 21)	XP_002276853.1	−3.52
495	pyruvate kinase isozyme A, chloroplastic isoform 1 (4, 11)	XP_002279975.1	−3.79
557	dihydrolipoyllysine-residue acetyltransferase component of pyruvate dehydrogenase complex, mitochondrial-like (8, 11)	XP_002282287.1	−4.11
194	glucose-6-phosphate 1-dehydrogenase, chloroplastic (7, 30)	XP_002266930.1	−19.38
*Cell Wall* (10)			
357	probable xyloglucan endotransglucosylase/hydrolase protein B (10)	XP_002274520.1	11.89
222	UDP-glucose 4-epimerase GEPI48 (10)	XP_002268294.2	7.31
34	hypothetical protein VITISV_001144 (m.: 10)	CAN61024.1	3.39
129	beta-xylosidase/alpha-L-arabinofuranosidase 2-like (10, 33)	XP_002264183.2	−2.07
561	probable rhamnose biosynthetic enzyme 1 (10)	XP_002282339.1	−2.12
772	UDP-sugar pyrophospharylase isoform 2 (10)	XP_003634394.1	−2.37
508	pectinesterase 2 (10)	XP_002280446.1	−3.32
12	acetyl-CoA carboxylase carboxyltransferase beta subunit (10, 11, 29)	ABE47543.1	d.
345	probable xyloglucan endotransglucosylase/hydrolase protein 30-like (10)	XP_002273975.1	d.
*Lipid Metabolism* (11)			
253	putative esterase HI_1161 isoform 1 (11)	XP_002269958.1	new
219	phospholipase D alpha 1 (11, 27)	XP_002268195.1	2.44
152	3-oxoacyl-[acyl-carrier-protein] synthase I, chloroplastic-like (11)	XP_002265207.1	−2.23
451	biotin carboxyl carrier protein of acetyl-CoA carboxylase-like (11)	XP_002278151.2	−2.64
216	3-oxoacyl-[acyl-carrier-protein] reductase, chloroplastic (11, 26)	XP_002268080.1	−3.72
737	3-oxoacyl-[acyl-carrier-protein] synthase 3 A, chloroplastic-like (11)	XP_003631438.1	−5.47
623	acyl-CoA-binding domain-containing protein 4-like isoform 1 (11)	XP_002284019.1	−5.81
648	probable linoleate 9S-lipoxygenase 5 (11)	XP_002284535.2	−7.06
414	biotin carboxyl carrier protein of acetyl-CoA carboxylase 1, chloroplastic (11, 18)	XP_002276955.2	d.
*N and amino acid metabolism* (12, 13)			
516	arginase-like (13)	XP_002280690.2	new
210	alanine--glyoxylate aminotransferase 2 homolog 2, mitochondrial (13, 19)	XP_002267787.1	5.42
178	methylmalonate-semialdehyde dehydrogenase [acylating], mitochondrial (13)	XP_002266390.1	5.29
170	adenosylhomocysteinase isoform 1 (13)	XP_002266154.1	2.99
787	phosphoserine aminotransferase, chloroplastic-like (13, 27)	XP_003635669.1	−2.04
763	3-phosphoshikimate 1-carboxyvinyltransferase, chloroplastic-like (13)	XP_003633923.1	−3.63
428	bifunctional 3-dehydroquinate dehydratase/shikimate dehydrogenase, chloroplastic-like (13)	XP_002277395.2	−4.68
25	glutamine synthetase (12)	CAA63982.1	−8.64
177	probable S-adenosylmethionine-dependent methyltransferase At5g37990-like (13, 17)	XP_002266288.2	−30.63
259	hydroxyacylglutathione hydrolase 3, mitochondrial-like (13)	XP_002270140.1	d.
*Secondary metabolism* (16)			
230	anthocyanidin 5,3-O-glucosyltransferase (16, 26)	XP_002268560.1	new
766	caffeic acid 3-O-methyltransferase 1-like isoform 1 (16, 26)	XP_003634161.1	new
678	probable NAD(P)H-dependent oxidoreductase 1 (3, 16)	XP_002285219.1	12.08
90	chalcone-flavonone isomerase 1 (16, 27)	P51117.1	3.07
206	anthocyanidin 5,3-O-glucosyltransferase-like (16, 26)	XP_002267573.1	2.44
135	stilbene synthase 1 (16)	XP_002264455.1	2.32
497	chalcone--flavonone isomerase-like isoform 1 (16)	XP_002280158.1	−2.09
427	probable cinnamyl alcohol dehydrogenase 1-like (16)	XP_002277375.1	−2.88
692	probable cinnamyl alcohol dehydrogenase 1 (16)	XP_002285406.1	−3.02
574	1-deoxy-D-xylulose 5-phosphate reductoisomerase, chloroplastic (16)	XP_002282761.1	−6.95
247	isoeugenol synthase 1 (16)	XP_002269639.1	d.
670	4-hydroxy-3-methylbut-2-en-1-yl diphosphate synthase (16)	XP_002285130.1	d.
*Hormone metabolism* (17)			
471	auxin-induced protein PCNT115 isoform 1 (17, 26)	XP_002278850.1	18.40
121	linoleate 13S-lipoxygenase 2–1, chloroplastic (17)	XP_002263854.1	3.85
515	gibberellin 3-beta-dioxygenase 3 (17, 26)	XP_002280670.1	d.
662	gibberellin 20 oxidase 3-like (16, 17, 26)	XP_002284983.1	d.
*Stress* (20)			
124	stress-related protein-like (16)	XP_002263944.1	new
715	disease resistance response protein 206 (20)	XP_002285676.1	new
581	osmotin-like protein OSM34 (20)	XP_002282917.1	3.47
653	MLP-like protein 34 (20)	XP_002284578.1	7.22
525	osmotin-like protein (20)	XP_002281193.1	5.06
91	basic endochitinase (20)	P51613.1	2.93
687	topless-related protein 4-like isoform 1 (20, 33)	XP_002285341.2	2.45
10	chitinase class I basic (20)	ABD64684.1	2.38
172	putative germin-like protein 2–1 (12, 20, 27, 30, 34)	XP_002266227.1	2.38
254	endoplasmin homolog (20)	XP_002270014.2	2.33
649	MLP-like protein 28-like isoform 1 (20)	XP_002284538.1	2.17
652	MLP-like protein 28 (20)	XP_002284570.1	2.10
359	major allergen Pru av. 1 (20, 27, 34)	XP_002274617.1	2.08
780	heat shock cognate protein 80-like (20)	XP_003635036.1	2.01
346	major allergen Pru ar 1 (20, 27, 34)	XP_002273982.1	−2.45
643	germin-like protein subfamily 1 member 17 (20, 27, 34)	XP_002284436.1	−2.82
334	germin-like protein 9–3 (15, 20)	XP_002273554.1	−3.62
195	putative germin-like protein 2–1 (12, 20, 27, 30, 34)	XP_002266984.1	−4.25
453	germin-like protein 9–3 (15, 20)	XP_002278170.1	−4.76
537	chitinase 2 (20)	XP_002281729.1	−4.80
102	pathogen-related protein (20)	XP_002263121.1	−7.46
74	unnamed protein product (m.: 11, 20)	CBI28159.3	d.
583	putative endo-1,3(4)-beta-glucanase 2-like (20)	XP_002282971.1	d.
*Redox* (21)			
8	catalase (21)	AAL83720.1	2.91
714	glutathione reductase, cytosolic (21)	XP_002285672.1	2.70
607	peroxiredoxin-2E, chloroplastic (21)	XP_002283652.1	−2.02
*Miscellaneous enzyme families* (26)			
193	glutathione transferase GST 23-like isoform 1 (26, 28, 33)	XP_002266900.1	8.62
777	probable glutathione S-transferase parC-like isoform 2 (26)	XP_003634746.1	4.78
15	glutathione S-transferase (26)	ABL84692.1	2.08
287	tropinone reductase 1-like (26)	XP_002271432.1	−2.14
274	epoxide hydrolase 2 (26)	XP_002270883.2	−2.40
323	glutathione S-transferase zeta class-like isoform 1 (26)	XP_002273077.1	−2.59
238	glutathione S-transferase U9 (26)	XP_002269118.1	−2.93
236	probable glutathione S-transferase (26)	XP_002268911.1	−3.06
674	NADP-dependent alkenal double bond reductase P1 (26, 34)	XP_002285167.1	−3.38
417	l-Ala-D/L-Glu epimerase (26)	XP_002277056.1	−3.45
412	UDP-glycosyltransferase 85A1-like (17, 26, 29)	XP_002276823.1	−3.84
197	momilactone A synthase (26)	XP_002267041.1	−4.51
320	momilactone A synthase-like (26)	XP_002272981.1	−5.53
127	glutathione transferase GST 23 (26)	XP_002264054.1	−7.67
536	glutathione S-transferase U7 isoform 1 (26, 28, 33)	XP_002281654.1	−9.51
512	probable glutathione S-transferase (26, 28, 33)	XP_002280532.1	−16.45
527	UDP-glycosyltransferase 83A1 (26)	XP_002281262.1	d.
748	epoxide hydrolase 2-like (26)	XP_003632381.1	d.
*DNA/RNA* (27, 28)			
207	DEAD-box ATP-dependent RNA helicase 37-like (27)	XP_002267581.2	31.87
235	DEAD-box ATP-dependent RNA helicase 56 (27, 28)	XP_002268833.1	11.78
148	transcription factor BTF3 (27, 34)	XP_002265041.1	4.42
676	polyadenylate-binding protein 2 (27)	XP_002285190.1	−2.62
502	ribonuclease UK114-like (27)	XP_002280251.1	−2.90
39	hypothetical protein VITISV_017556 (m.: 27)	CAN66609.1	−3.96
205	uncharacterized protein LOC100253093 (m.: 27, 29, 34)	XP_002267536.1	−5.72
42	hypothetical protein VITISV_020351 (probable ADP-ribosylation factor GTPase-activating protein AGD11) (27)	CAN67438.1	d.
133	KH domain-containing protein At4g18375 isoform 1 (27)	XP_002264417.1	d.
240	poly(rC)-binding protein 3-like (27)	XP_002269249.1	d.
*Protein* (29)			
180	miraculin (20, 29, 31)	XP_002266430.1	11.55
651	26S proteasome non-ATPase regulatory subunit 14 (29)	XP_002284566.1	4.76
387	probable protein phosphatase 2C 58 (29)	XP_002275890.1	3.85
137	26S proteasome non-ATPase regulatory subunit 4 (29)	XP_002264558.1	2.95
352	60S ribosomal protein L10a-1-like (29)	XP_002274218.2	2.73
696	60S ribosomal protein L23-like (29)	XP_002285443.1	2.69
213	uncharacterized protein At2g37660, chloroplastic (m.: 29)	XP_002267965.1	2.55
190	elongation factor 2-like isoform 1 (29)	XP_002266780.1	2.40
266	60S ribosomal protein L11–1-like (29)	XP_002270266.1	2.34
303	serine carboxypeptidase-like 18 (29)	XP_002272116.1	2.27
604	probable glutamate carboxypeptidase 2-like isoform 1 (27, 29)	XP_002283565.2	2.20
57	hypothetical protein VITISV_003812 (60S acidic ribosomal protein P0) (29)	CAN80537.1	2.16
543	heme-binding protein 2-like (19, 29)	XP_002281829.1	2.10
538	cucumisin-like (29)	XP_002281790.2	−2.07
559	serine carboxypeptidase II-3-like (29)	XP_002282331.1	−2.38
572	ATP-dependent Clp protease proteolytic subunit 4, chloroplastic (29)	XP_002282652.1	−2.43
169	60S acidic ribosomal protein P2B isoform 1 (29)	XP_002266030.1	−2.60
2	40S ribosomal protein SA (29)	A5BUU4.1	−3.26
501	eukaryotic translation initiation factor 3 subunit M (29)	XP_002280247.1	−4.72
633	uncharacterized protein LOC100262703 (dipeptidyl-peptidase 5) (29)	XP_002284264.1	−4.78
616	ADP-ribosylation factor-like protein 5 (29)	XP_002283837.1	−4.88
702	probable protein phosphatase 2C 60 (29)	XP_002285549.1	−4.97
232	protein transport protein SEC23-like (29)	XP_002268633.2	−17.96
518	pyrrolidone-carboxylate peptidase isoform 4 (29)	XP_002280794.1	d.
577	subtilisin-like protease (29)	XP_002282841.1	d.
578	protein transport protein Sec24-like At3g07100-like (29)	XP_002282857.1	d.
620	aspartic proteinase nepenthesin-1-like (29)	XP_002283889.2	d.
*Cell / signaling / development* (30, 31, 33)			
114	70 kDa peptidyl-prolyl isomerase (31)	XP_002263566.2	new
250	11S globulin seed storage protein 2 (28, 33)	XP_002269868.1	50.05
94	peptidyl-prolyl cis-trans isomerase H (28, 33)	XP_002262773.1	25.35
490	PRA1 family protein F2-like (30, 31)	XP_002279772.1	13.62
741	coatomer subunit gamma-2-like (31)	XP_003631645.1	5.84
644	glutelin type-A 1 (28, 33)	XP_002284459.1	5.53
685	probable plastid-lipid-associated protein 6, chloroplastic (31)	XP_002285326.1	2.21
45	hypothetical protein VITISV_0120489 (plastid lipid-associated protein) (31)	CAN69132.1	2.15
391	tubulin alpha chain (31)	XP_002275973.1	−2.05
503	syntaxin-71 (27, 31)	XP_002280272.1	−3.77
459	PITH domain-containing protein At3g04780 (33)	XP_002278320.1	d.
*Transport* (34)			
296	V-type proton ATPase subunit H-like (34)	XP_002271887.1	5.21
365	V-type proton ATPase subunit E (29, 34)	XP_002274995.1	3.68
358	probable aquaporin PIP2–5 (34)	XP_002274555.1	−3.94
*Others* (15, 18, 23, 24)			
418	ferritin-3, chloroplastic (15)	XP_002277114.1	new
196	selenium-binding protein 1 (15)	XP_002267004.1	10.44
143	soluble inorganic pyrophosphatase-like (23)	XP_002264695.2	2.20
576	nicotinate phosphoribosyltransferase-like (18, 23)	XP_002282786.1	−6.86
433	probable carboxylesterase 15 (24)	XP_002277507.1	−9.08
332	uracil phosphoribosyltransferase (23)	XP_002273489.1	d.
*Hypothetical / Unknown function* (35)			
108	uncharacterized protein At5g48480 (35)	XP_002263284.1	20.80
733	probable nucleoredoxin 1-like (35)	XP_003631263.1	8.46
582	elicitor-responsive protein 3 (35)	XP_002282926.1	4.99
80	unnamed protein product (35)	CBI34823.3	4.73
771	uncharacterized protein LOC100854733 (35)	XP_003634361.1	3.22
98	CBS domain-containing protein CBSX3, mitochondrial isoform 1 (35)	XP_002262902.1	2.02
580	uncharacterized protein LOC100259086 (35)	XP_002282908.1	−2.08
281	nodal modulator 1 (35)	XP_002271147.1	−2.24
443	transmembrane protein 111 (35)	XP_002277989.1	−2.43
343	uncharacterized protein LOC100242710 (35)	XP_002273917.1	−3.60
164	uncharacterized protein LOC100265424 (D-alanine--D-alanine ligase family protein) (35)	XP_002265851.1	−3.64
335	S-norcoclaurine synthase (35)	XP_002273566.1	−4.27
48	hypothetical protein VITISV_002394 (35)	CAN70694.1	−4.82
138	non-lysosomal glucosylceramidase (35)	XP_002264575.2	−5.50
466	clavaminate synthase-like protein At3g21360 (35)	XP_002278552.1	−6.68
40	hypothetical protein VITISV_001156 (35)	CAN67361.1	d.
547	GDT1-like protein 4 (35)	XP_002281939.1	d.

Numbers reported in brackets refer to bin code (i.e. major functional categories). n.: identification number. f.c.: bin code of functional categories. Name: for proteins without a name in brackets are indicated the results from BLAST alignment against NCBI *Viridiplantae* database; m.: classification obtained through grape/Arabidopsis or grape/potato matching by BLASTp algorithm (E.value < 10^–^20). Δ: fold changes in WS plants with respect to the Control ones (up: %(SI)WS/%(SI)C, down: - %(SI)C/%(SI)WS). new: not present in C; d.: disappeared, not present in WS

**Fig. 1:**
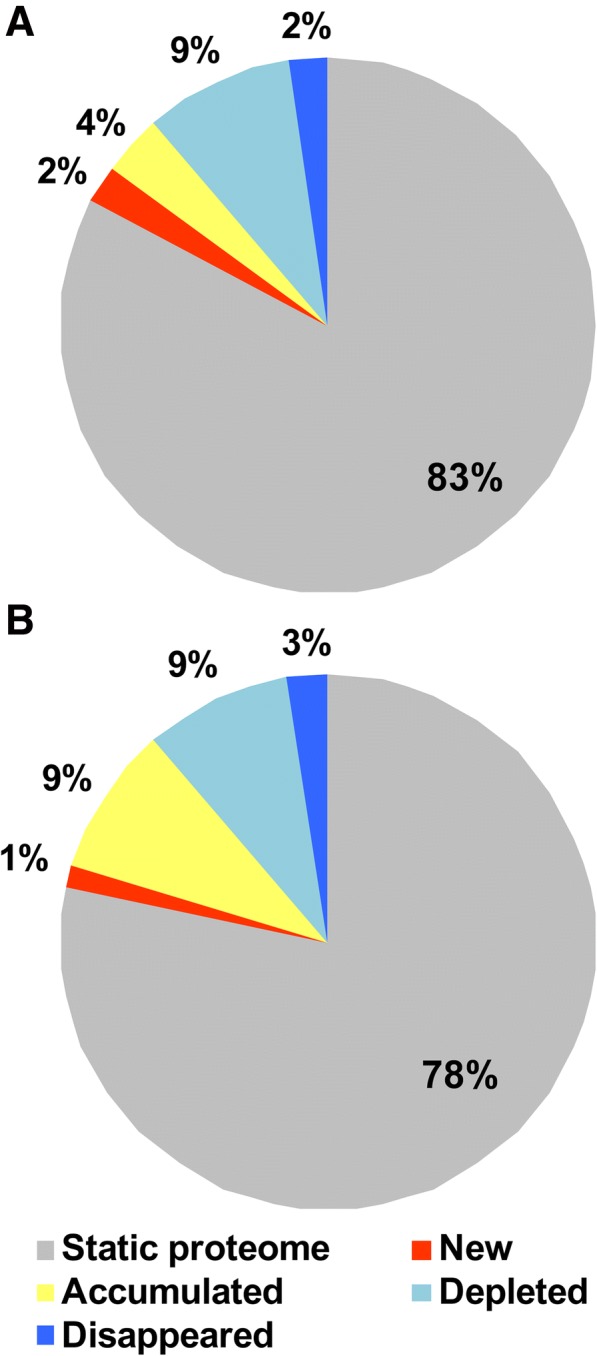
Proteomic changes in response to water stress in 101.14 (**a**) and M4 (**b**) rootstock genotypes. Proteins that did not show an at least two-fold change between control and WS (Student’s t-test; *p* < 0.05) were classified as “static proteome”. Proteins that significantly changed were divided into four groups: new, accumulated, depleted and disappeared

The majority of the identified proteins did not change in abundance under WS, and this was named the “static proteome” (Fig. 1). In detail, the static proteome was 83 and 78% in the 101.14 and the M4 rootstock genotypes, respectively (Fig. 1a and b). Proteins that changed in abundance in WS were 181 and 186 for 101.14 and M4, respectively. Among these, 48 were identified only in 101.14, whereas 64 were found only in M4 (Additional file 3: Table S3 A, B and C). Small differences occurred between proteins that appeared (named “new”, 2 and 1% in 101.14 and M4, respectively) or disappeared (2 and 3% in 101.14 and M4, respectively) in WS. Moreover, 9% of the proteins decreased in abundance in both genotypes, while the accumulated proteins were 4 and 9% in 101.14 and M4, respectively (Fig. 1a and b).

**Fig. 2:**
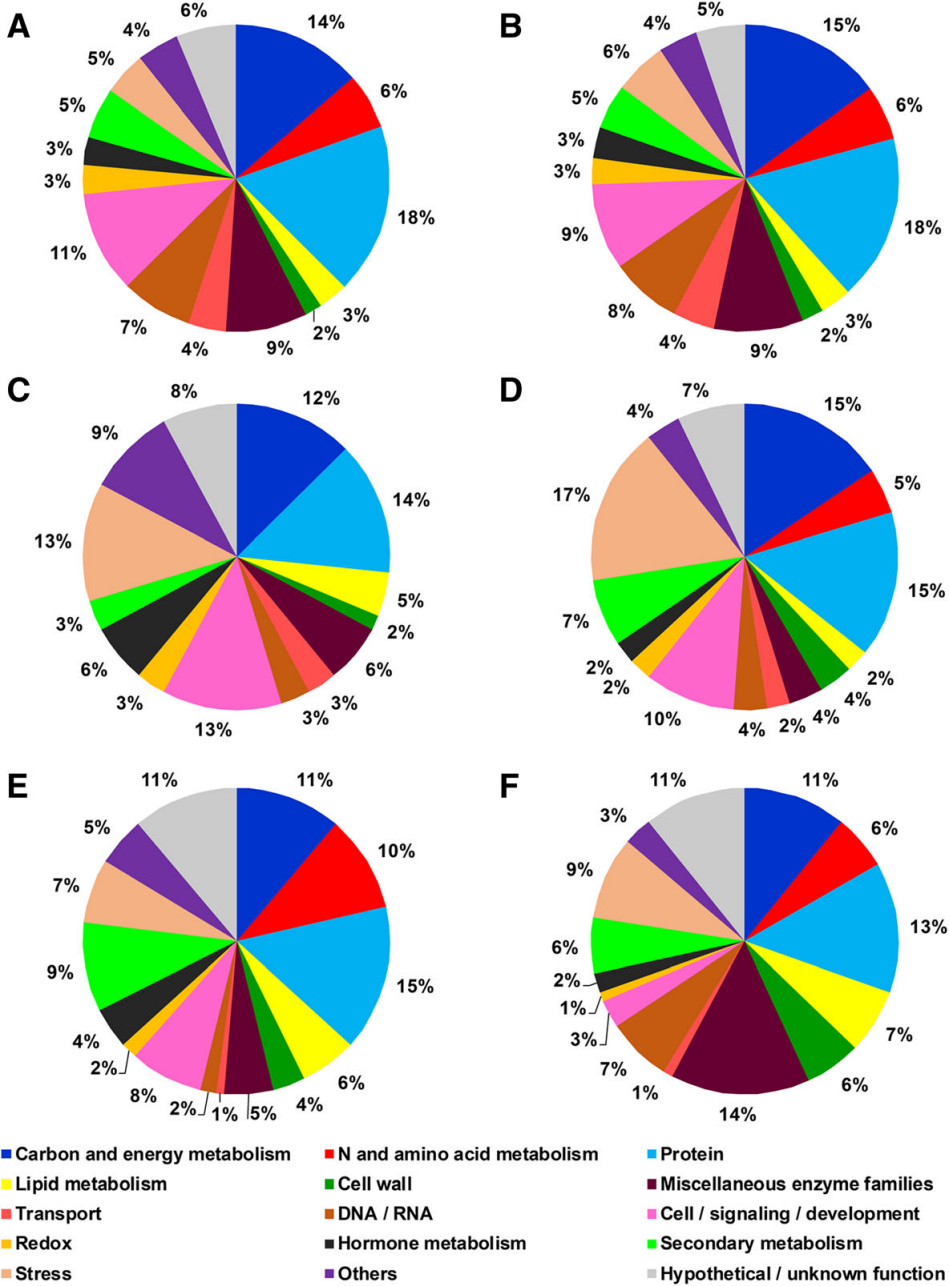
Functional distribution of identified proteins in the 101.14 (**a**, **c** and **e**) and M4 (**b**, **d** and **f**) rootstock genotypes. **a**, **b** distribution of all the proteins identified in the control condition. **c** and **d** proteins that increased in abundance/appeared in WS. **e** and **f** proteins that decreased in abundance/disappeared in WS

Functional classification was made according to the bin hierarchical tree developed by MapMan ontology [34] using a *Vitis vinifera* mapping file (see Methods for details; Additional file 3: Table S3 A and B for the data). In the control condition, the two rootstock genotypes showed a very similar functional distribution of the identified proteins (Fig. 2a and b for 101.14 and M4, respectively). About 50% of these fell into four functional categories: carbon and energy metabolism, protein, miscellaneous enzyme families and stress. Considering only the proteins that changed significantly in abundance, essentially all the functional categories were affected by WS, but the extent of these changes was very different in the two genotypes (Fig. 2c, d, e and f). Proteins that increased/appeared (64 and 84 for 101.14 and M4, respectively) were represented in all the functional categories. Interestingly, the 101.14 genotype did not show any increase of proteins involved in N and amino acid metabolism. Comparing the responses between the two genotypes, 101.14 showed a greater number of increased/appeared proteins concerned to lipid metabolism, hormone metabolism, miscellaneous enzyme families and others, while for all the remaining functional categories the number of accumulated proteins was generally higher in M4 (Fig. 2c and d for 101.14 and M4, respectively). An opposite response occurred for proteins that decreased/disappeared in WS, which were 117 and 102 for 101.14 and M4, respectively. Indeed, only in four functional categories, i.e., cell wall, stress, miscellaneous enzyme families and DNA/RNA, the number of proteins that decreased in abundance was higher in M4, while for essentially all the remaining categories the higher number was found in 101.14 (Fig. 2e and f for 101.14 and M4, respectively).

### Main metabolic pathways of the primary metabolism

**Fig. 3:**
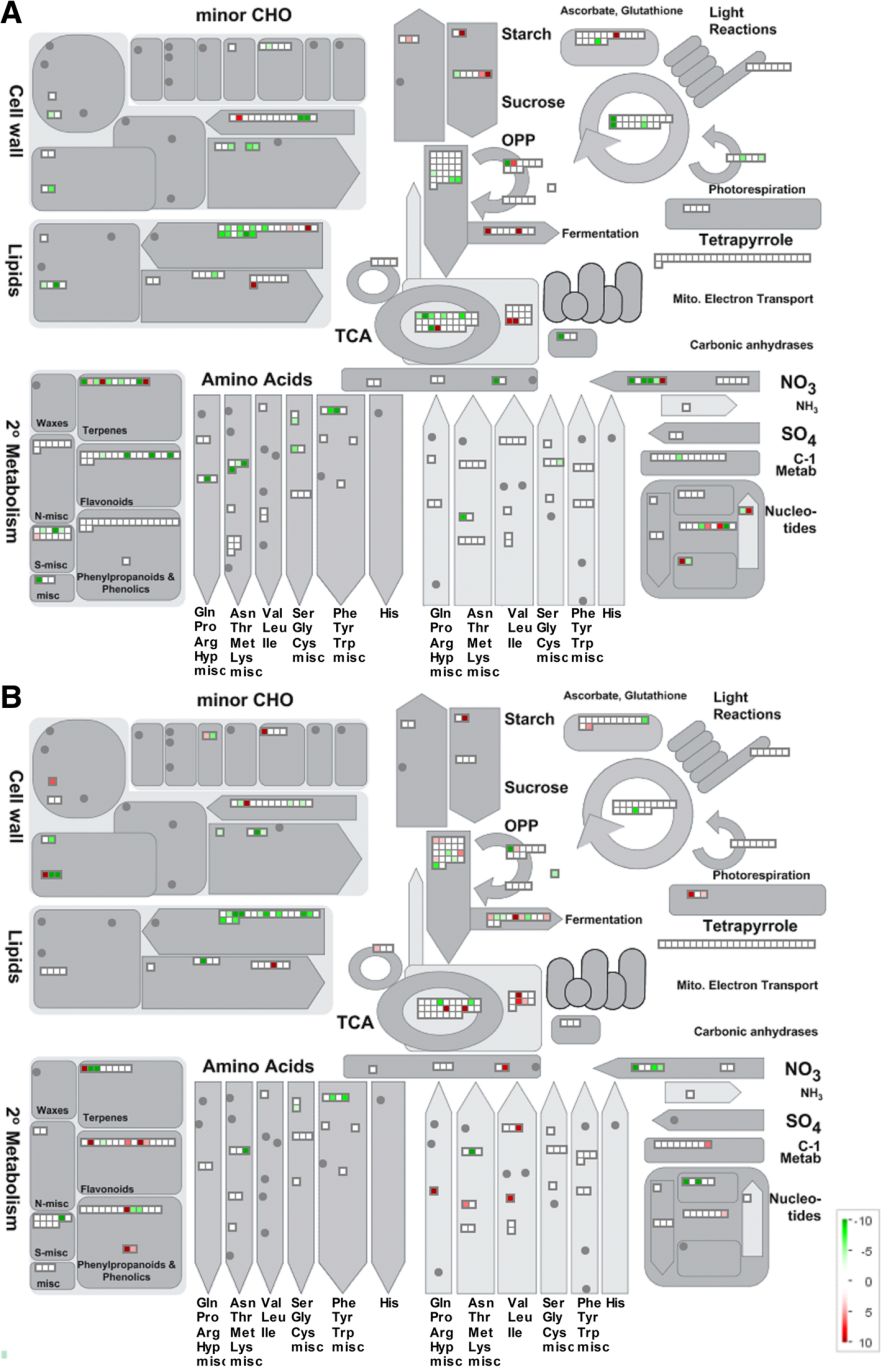
MAPMAN overview of metabolic pathways in WS in root tissue of 101.14 (**a**) and M4 (**b**) rootstock genotypes. Green circles: decrease, white circles: no change, red circles: increase in protein abundance in WS respect to the control (see colour scale)

**Fig. 4:**
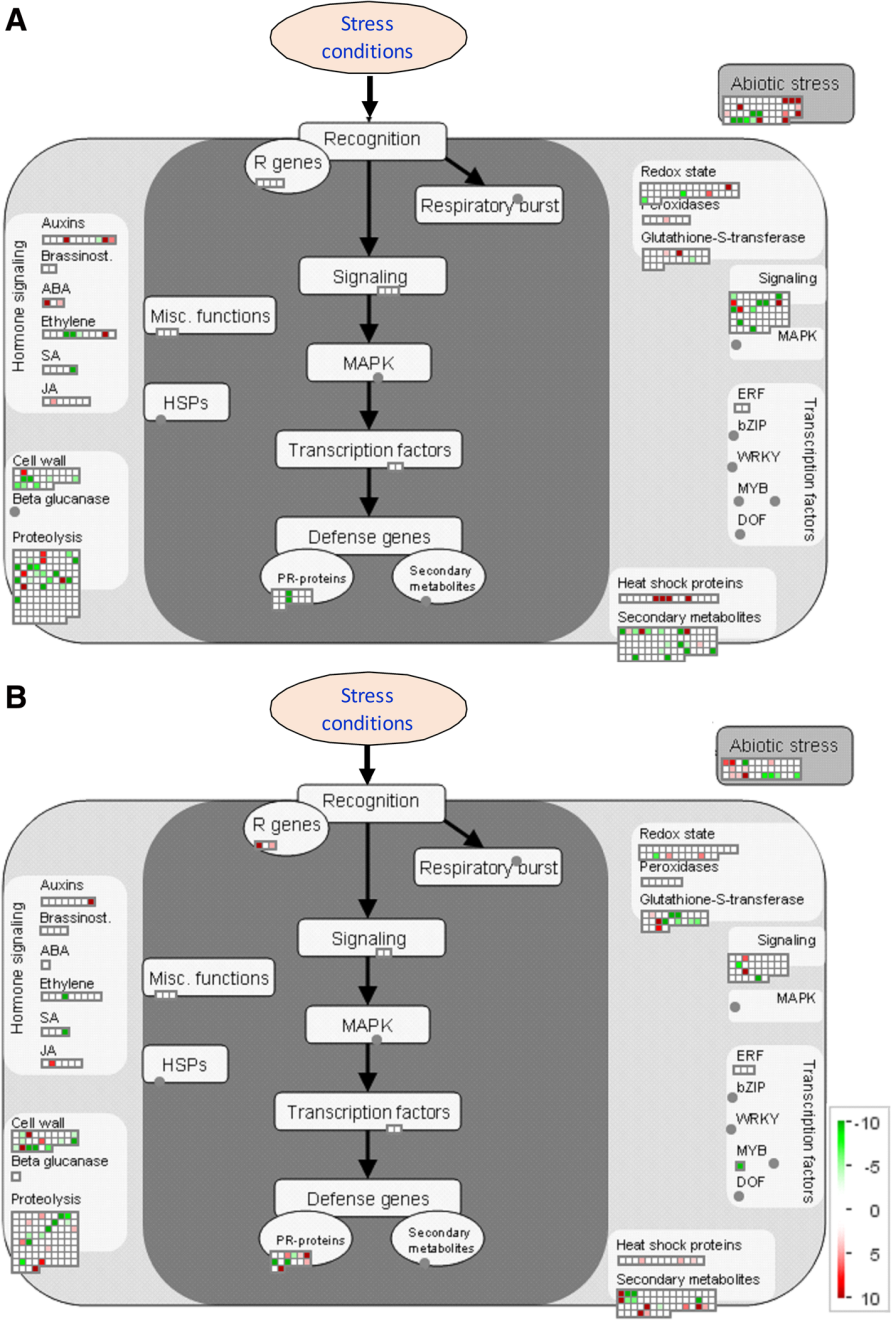
MAPMAN overview of stress pathways in WS in root tissue of 101.14 (**a**) and M4 (**b**) rootstock genotypes. Green circles: decrease, white circles: no change, red circles: increase in protein abundance in WS respect to the control (see colour scale)

The analysis of the nature of the proteins affected by WS highlighted changes in many metabolic pathways with particular differences between the two genotypes (Tables 1 and 2). For a better visualization, datasets containing all of the proteins identified in each genotype were used to produce heat maps concerning the overview of both the main metabolic pathways (Fig. 3) and those known to be involved in stress responses (Fig. 4).

WS led to deep changes in the functional classes of carbon and energy metabolism (Tables 1 and 2; Fig. 3; Additional file 3: Table S3 C). In both rootstock genotypes, enzymes involved in starch mobilization (alpha-1,4 glucan phosphorylase L isozyme) or in the sucrose biosynthetic pathway (phosphoglucomutase, UTP-glucose-1-phosphate uridylyltransferase and glucose-6-phosphate isomerase) were found. On the basis of the observed changes in abundance, M4 showed a greater activation of these pathways. Moreover, an increase in sucrose synthase 2, an enzyme involved in the degradation of this sugar, occurred only in 101.14.

Many of the changing proteins classified in the cell wall functional class decreased in abundance in WS (Tables 1 and 2; Figs. 3 and 4). Nevertheless, UDP-glucose 4-epimerase GEPI48, an enzyme that probably plays a central role in biosynthesis and growth of cell walls [35], rose during WS in both genotypes. Moreover, a xyloglucan endotransglucosylase/hydrolase protein B, an enzyme involved in wall loosening [28], showed an evident enhancement only in M4. In this genotype, the hypothetical protein VITISV_001144 (CAN61024.1), that shows a high similarity with some leucine-rich repeat (LRR) proteins, also surged up under WS.

Many plastidial enzymes, such as triosephosphate isomerase, enolase 1, pyruvate kinase isozyme A, ferredoxin-NADP reductase and glucose-6-phosphate 1-dehydrogenase, decreased in abundance under WS in both genotypes. Unlike the plastid isoform, the abundance of cytosolic glucose-6-phosphate dehydrogenase increased in both genotypes (Tables 1 and 2).

Some units of the pyruvate dehydrogenase complex (dihydrolipoyl dehydrogenase, E1 component subunit beta and dihydrolipoyllysine-residue acetyltransferase) reduced their abundance in WS in both genotypes, although the effect was more evident in 101.14. Moreover, only in M4, there was an increase in phosphoenolpyruvate carboxylase, which participates in replenishing TCA cycle intermediates [36].

Several enzymes of the TCA cycle were affected by WS (Fig. 3). In the 101.14 genotype, an ATP-citrate synthase and a fumarate hydratase increased in abundance, whilst a succinate dehydrogenase iron-sulfur subunit 1 disappeared. In M4, in addition to the same ATP-citrate synthase, an isocitrate dehydrogenase and a 2-oxoglutarate dehydrogenase also showed increases during water stress (Tables 1 and 2).

Among the proteins classified in carbon metabolism there was an aldehyde dehydrogenase family 7 member A1 that increased in abundance in WS in both genotypes (Tables 1 and 2). This protein was previously associated to the osmotic adaptation response to different abiotic stresses or ABA treatment [37, 38].

Only in M4 there was a surge in abundance of a succinate-semialdehyde dehydrogenase (acetylating) and a formate dehydrogenase (mitochondrial). The first of these is known to be involved in the metabolism of γ-aminobutyric acid (GABA) and plays a very important role in the response to reactive oxygen species (ROS), as suggested by the behaviour of the *Arabidopsis ssadh* mutant that accumulates elevated levels of H_2_O_2_ and is sensitive to UV-B light and heat stresses [37]. Similarly, an involvement of mitochondrial formate dehydrogenase under stress conditions, such as drought and cold, was previously suggested to be linked to the requirement to metabolize the increasing levels of formate and to use this alternative substrate to sustain the requirement for reducing power [39].

Among the proteins involved in lipid metabolism, a phospholipase D alpha 1 increased in WS in both genotypes. This enzyme, which produces phosphatidic acids hydrolyzing membrane glycerol-phospholipids, plays an important role in the responses that involve abscisic acid [40]. Many of the other changing proteins classified in this functional class, such as the carboxyl carrier protein of acetyl-CoA carboxylase-like, 3-oxoacyl-[acyl-carrier-protein] synthase I and acyl-CoA-binding domain-containing protein 4-like isoform 1, which are involved in the metabolism of fatty acids, decreased in WS. Only in 101.14, glyoxysomal fatty acid beta-oxidation multifunctional protein MFP-a, involved in fatty acid degradation, showed a very large increase in abundance in WS (Table 1). In this unfavourable condition, 101.14 also induced a long chain acyl-CoA synthetase.

In both genotypes, WS negatively affected many proteins classified in the functional class of N and amino acid metabolism (Fig. 3). Nevertheless, the results revealed that the decrease in abundance of enzymes involved in amino acid (aspartate, serine, glycine, cysteine and aromatic amino acids) biosynthesis was more pronounced in 101.14. Moreover, only in M4 some of these proteins increased in abundance/appeared during WS. Among these was an arginase-like, known to be involved also in proline metabolism [41].

### Secondary metabolism and miscellaneous enzyme families

Differences between the two genotypes were detected among the proteins classified in the secondary metabolism functional class (Tables 1 and 2; Fig. 4). In 101.14, 2-C-methyl-D-erythritol 2,4-cyclodiphosphate synthase, involved in the MEP/DOPX pathway [42], appeared in WS, whilst all the other changing proteins of this class decreased. However, in M4 a minor number of decreasing proteins was found, whereas six proteins involved in secondary metabolism rose or appeared in WS. Among these there were enzymes involved in the biosynthesis of flavonoids (chalcone-flavonone isomerase 1; anthocyanidin 5,3-O-glucosyltransferase) and stilbenes (stilbene synthase 1).

Many of the changing proteins classified in the miscellaneous enzyme families were identified as glutathione S-transferases (GST). Among these, only one was referred to the same form and decreased in both genotypes, while the other WS-affected GSTs were assigned to different entries. In M4, three GSTs increased and six decreased in WS. In 101.14 only three GSTs changed their levels. Among the proteins classified in the miscellaneous enzyme category that surged up in WS, a peroxidase 3 was found only in 101.14. Moreover, an NADP-dependent alkenal double bond reductase P1, which catalyzes the reduction of the α,β-unsaturated bond of the reactive carbonyls, playing an important role in the antioxidative defence mechanisms [43], dropped in WS in 101.14, while in M4 the reduction was much lesser severe (− 4643% and − 238% in 101.14 and M4, respectively).

### Hormone and redox metabolisms

A few proteins of which the levels varied in WS belong to the hormone metabolism functional class, especially in M4 (Tables 1 and 2; Fig. 4). Among the proteins that increased in both genotypes were the linoleate 13S-lipoxygenase 2–1, involved in the biosynthesis of jasmonic acid [44] and the auxin-induced protein PCNT115 isoform 1. The extent of the observed changes was very different in the two genotypes: + 155 and + 193% in 101.14, and + 285% and + 1740% in M4 respectively. Nevertheless, only in 101.14 was there an increase in an isoform of auxin-repressed 12.5 kDa protein as well as a decrease in indole-3-acetic acid-amido synthetase GH3.1. In both genotypes, WS negatively affected the abundance of the gibberellin 3-beta-dioxygenase and of the gibberellin 20 oxidase 3, both involved in gibberellin biosynthesis [45].

Only in 101.14, there was an increase in HVA22-like protein a, known to be induced by ABA under different abiotic stresses [46], while 1-aminocyclopropane-1-carboxylate oxidase 5, an enzyme that catalyzes the last reaction of ethylene biosynthesis, disappeared.

Similarly, proteomic analysis revealed differences between the two genotypes concerning a few enzymes involved in the redox metabolism (Tables 1 and 2; Fig. 4). In detail, a peroxiredoxin-2E decreased to similar extent in both genotypes, whereas a catalase and a glutathione reductase increased in WS only in M4, and another catalase decreased in stress conditions only in 101.14. In this last genotype the glutathione S-transferase DHAR3, a chloroplastic enzyme involved in the scavenging of ROS [47], also increased.

### Nucleic acid and protein metabolisms

Among the proteins relating to DNA/RNA functionalities, the basal transcription factor 3 (BFT3) rose in WS only in M4 (Tables 1 and 2). In this genotype an upturn in two proteins involved in RNA metabolism (DEAD-box ATP-dependent RNA helicase 37-like and DEAD-box ATP-dependent RNA helicase 56) also occurred. At the same time, the appearance of a ribonuclease 3 and of a proactivator polypeptide-like 1 isoform 1 occurred only in 101.14 (Tables 1 and 2).

In WS conditions, some proteins involved in protein synthesis or degradation significantly varied in abundance (Tables 1 and 2; Fig. 4). The results showed up the differences between the two genotypes. Among the proteins that increased, some ribosomal proteins (i.e., a 60S ribosomal protein L10–1-like, a 60S ribosomal protein L11–1-like, a 60S ribosomal protein L23-like and a 60S acidic ribosomal protein P0), as well as the elongation factor 2-like isoform 1, were observed only in M4. Nevertheless, other proteins belonging to this functional class, such as the 60S acidic ribosomal protein P2B isoform 1, the translation initiation factor 3 subunit M and the 40S ribosomal protein SA, decreased in WS in this genotype. In 101.14, WS induced a rise in a 40S ribosomal protein S15a-like isoform 2 and in the translation initiation factor 3 subunit E. In both genotypes, WS affected the abundance of proteins with diverse functions, from protein catabolism to protein maturation. Among these, in both genotypes there was an increase in 26S proteasome non-ATPase regulatory subunits. Moreover, in 101.14 a N-carbamoyl-L-amino acid hydrolase-like, a protease Do-like 1 chloroplastic-like, and an aspartic proteinase nepenthesin-1 increased, whereas in M4 an upturn of a serine carboxypeptidase-like 18 and a probable glutamate carboxypeptidase 2-like isoform 1 took place. Water stress negatively affected in both genotypes other enzymes with similar functions, such as aspartic proteinase nepenthesin-1-like, pyrrolidone-carboxylate peptidase isoform 4, serine carboxypeptidase II-3-like, subtilisin-like protease-like and cucumisin-like. The same trend was observed for a protein transport protein SEC24-like, a subunit of COPII coat vesicles [48]. Only in 101.14, in WS four proteins, identified as a serine carboxypeptidase-like 18, two serine carboxypeptidase II-3-like and a serine carboxypeptidase-like 45-like, decreased. To these enzymes, which belong to a larger class of proteases in plants, both proteolytic and non-proteolytic functions have been attributed [49].

### Other cell functions

Among the proteins belonging to the cell/signalling/development functional classes (Tables 1 and 2), a 70 kDa peptidyl-prolyl isomerase was identified. This protein, which appeared in WS in both genotypes, was previously described as changing its cellular localization under heat stress, according to its involvement in preserving cell functionality under abiotic stress [50]. The evident increase in the level of a peptidyl-prolyl cis-trans isomerase H taking place during WS was exclusive to M4. In the same condition, only in 101.14 the appearance of a transmembrane emp24 domain-containing protein and the upsurge of a VAMP-like protein YKT61-like (both involved in vesicle-mediated transport) were observed. Interestingly, two proteins of the PRA1 protein family (small transmembrane proteins controlling vesicle trafficking; Kamei et al. [51]) showed a mirror behaviour in the two genotypes: one disappeared in 101.14 (PRA1 family protein B4-like) while the other increased in M4 (PRA1 family protein F2-like). Moreover, a coatomer subunit gamma-2-like, a COP protein involved in the ER/Golgi network [52], increased only in M4. By contrast, the coatomer subunit epsilon-1 isoform 1 decreased in 101.14.

Water stress also affected a few cytoskeletal proteins (Tables 1 and 2). In particular, in 101.14 an actin-depolymerizing factor 10 was detected only in stressed roots, while in the same experimental condition a tubulin beta-1 chain isoform 1 decreased. Moreover, a tubulin alpha chain decreased in M4.

Water stress also affected the abundance of a few storage proteins (Tables 1 and 2). In detail, an upturn of a glutelin type-A 1 occurred in both genotypes, while only in M4 there was a consistent rise of an 11S globulin seed storage protein 2. Aquaporin TIP2.3 and PIP2–5 decreased in abundance in 101.14 and M4, respectively.

Among the proteins belonging to the transport functional class (Tables 1 and 2), WS induced in 101.14 the increase of a pyrophosphate-energized vacuolar membrane proton pump 1, whereas two subunits (H-like and E) of the V-type proton ATPase were positively affected by WS in M4.

### Proteins involved in stress responses

Water stress affected the abundance of 17 and 23 proteins belonging to the stress functional class in 101.14 and M4, respectively (Tables 1 and 2; Fig. 4). Some of these were identified as germin-like proteins (GLP), a group of proteins with heterogeneous functions belonging to the cupin superfamily known to change their levels in different biotic and abiotic stress conditions [53]. In both genotypes, two GLPs were positively affected by WS, while the other ones were found to decrease in this adverse condition, though to a major extent in 101.14.

Similar considerations also applied to both the stress-related and the major latex protein (MLP) like proteins. Although further work is necessary to clarify the specific function(s) of MLPs, emerging evidence suggests their role in improving the tolerance to stress conditions [54–56]. In detail, in 101.14 the rise of two stress-related protein-like and of two MLP-like proteins was observed. In M4 the same stress-related protein-like and MLP-like protein 34 appeared, and two other MLP-like proteins increased in abundance.

Two osmotin like-proteins, which play an important role in osmotic adjustment to tolerate WS conditions [57, 58], rose in abundance in WS only in M4. In the same genotype, WS also positively affected a disease resistance response protein 206, a chitinase class I basic, a basic endochitinase, an endoplasmin homolog, a topless-related protein 4-like isoform 1, and a pathogen-related protein. Nevertheless, only in 101.14, WS induced the appearance or a great increase in three heat-shock proteins.

### Metabolic analysis

**Fig. 5:**
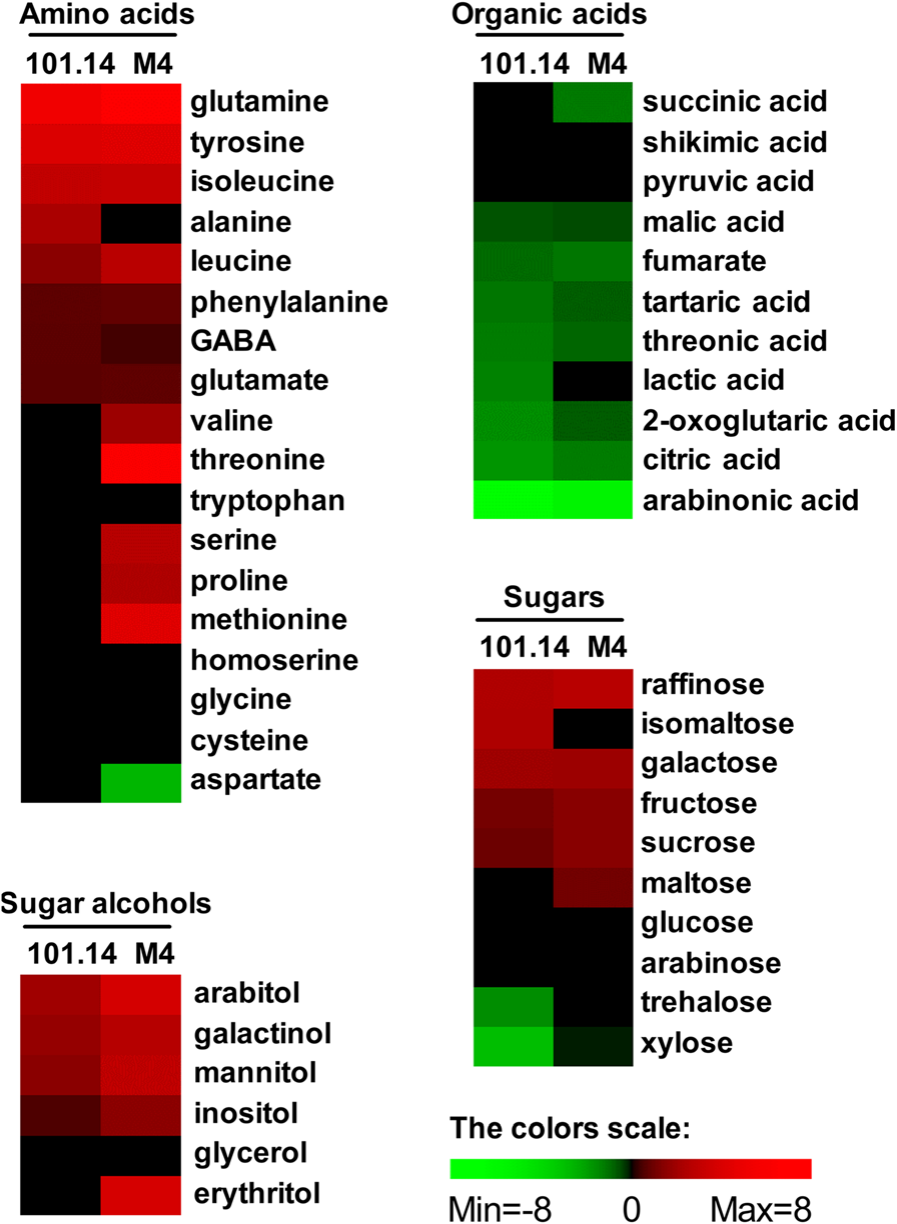
Effect of WS on metabolite levels in the root tissue of 101.14 and M4 rootstock genotypes. The clustering analysis was performed with PermutMatrix graphical interface after Z-score normalization of the values. Pearson’s distance and Ward’s algorithm were used for the analysis. Each coloured cell represents the value of the normalized WS/Control ratio, according to the colour scale at the bottom of the figure. Red increased levels, green: decreased levels. Black: identified metabolites not showing significant variation at the Student’s t-test (*p*-value < 0.05)

The changes of metabolite contents induced by WS are visualized in Fig. 5. Many metabolites appeared to be affected by the stress condition. Overall, amino acids, sugars and sugar alcohols were positively affected by WS, whilst almost all organic acids decreased under the unfavourable condition. Some of the changes were different in the two genotypes.

The analysis revealed in both genotypes a quite similar increasing trend, during WS, for some sugars, such as raffinose, galactose, fructose and sucrose. The increase of the last two metabolites was higher in M4. However, WS induced a greater increase in maltose in M4 and higher levels of iso-maltose in 101.14.

WS raised the levels of some sugar alcohols, such as inositol, galactol, mannitol and arabitol in both genotypes, but they increased to a higher extent in M4. Moreover, an upturn of erythritol occurred only in this last genotype.

Under WS, the levels of many metabolites of the Krebs cycle were negatively affected in both genotypes while, among the amino acids, similar increases in tyrosine, isoleucine, phenylalanine glutamine and glutamate took place. However, a greater increase in leucine was observed in M4. Only in 101.14 was an upsurge of alanine detected, whereas particular increments in valine, serine, methionine, threonine, and proline were measured in M4. Under WS, an increase of GABA level occurred in both genotypes, but to a major extent in 101.14.

## Discussion

According to previous studies conducted in the same experimental conditions [29, 30], proteomic analysis strengthens the crucial role of roots in the plant responses to water stress (WS) and allows some particular traits of WS tolerance in a perennial plant species such as grapevine to come to light. The physiological measurements previously performed by Meggio and co-workers, revealed different capabilities of 101.14 and M4 rootstock genotypes to sustain CO_2_ assimilation rate (A*n*) and stomatal conductance (g_s_) [29]. In both genotypes there occurred a progressive decrease of g_s_ and A*n* during the first days of stress, while afterwards 101.14 showed a further decrease whereas M4 partially recovered to 20 and 40% of the control condition for g_s_ and A*n*, respectively. This anisohydric behaviour also appeared linked to a better ability of roots to sustain loss of water [29]. When the field capacity was reduced to 30%, the roots of M4 showed, indeed, a greater capacity to adjust its osmolality adequately as well as to maintain cell integrity, as suggested by the protein and ion contents and by the recovery observed after re-watering [29].

The present work shows that the water-limiting condition induced significant changes in the whole root proteome (i.e., 17 and 22% of the quantified proteins in 101.14 and M4, respectively) and this result appeared to be well related to the capability of the genotype to respond more (M4) or less (101.14) positively to this stress condition. Whereas the functional distribution of the identified proteins in the control condition was quite similar in the two genotypes, important differences were found among the proteins that changed in WS (Fig. 2). The differences observed between the two genotypes were also consistent with the trends of the main metabolites (Fig. 5). Taken together, M4 showed positive metabolic responses potentially able to counteract the WS effects, whilst in 101.14 the suffering status of the roots became evident.

### Root growth and osmotic adjustment

An important feature observed in plants exposed to water-limiting conditions is their ability to maintain primary root elongation, a process that is strictly dependent on several different responses. Crucial aspects for sustaining cell growth are to guarantee an adequate cell wall extensibility as well as to maintain water uptake, which depends heavily on an adjustment of the solute potential [28, 59].

Proteomic analysis showed that WS induced in both genotypes changes in abundance of some proteins involved in the biosynthesis and expansion of cell walls, as well as higher levels of typical osmoprotective compounds (i.e., amino acids, raffinose and some sugar alcohols). M4 showed a better capability to respond to the adverse condition, as indicated by the increase in specific proteins involved in cell wall loosening (i.e. xyloglucan endotransglucosylase/hydrolase protein B). Moreover, even if both genotypes showed an increase in compatible solutes, this response was greater in M4, as suggested by the increased levels of proline and of many sugar alcohols. Recently, the physiological relevance of this last class of compounds in the response to WS was investigated in fruit and leaves of grapevine, where WS induced the synthesis of these osmoprotective solutes, especially in the fruit mesocarp [60]. Our study shows that WS also evoked the synthesis of some polyols, such as mannitol, inositol, galactinol, and erythritol, in the roots. Overall, the higher polyol levels detected in the tolerant genotype M4 confirmed the importance of these compounds in improving WS response suggested by previous works [60, 61]. Furthermore, other metabolites, such as amino acids, appeared to contribute to the osmotic adjustment evoked by WS with a more massive response in M4 (see below). In addition, it is interesting to observe that only in M4 was there found an increase in two osmotin like-proteins, a class of proteins previously found to increase WS tolerance [57, 58]. Taken together, our study highlights a greater capability of M4 to activate molecular and biochemical processes useful to sustain the osmotic adjustment needed for root growth in a very severe WS condition.

Even though previous works pointed out the central role of aquaporins in the water stress response, the present study found decreases of aquaporin TIP2–3 and PIP2–5 in 101.14 and M4, respectively. This ostensible discrepancy could be explained by observing that our study analysed the whole root organ, while the water channel play an important role only in the young roots [19, 24, 62]. At the same time, we should consider that these proteins show diurnal changes in expression [62]. The proteomic analysis was performed on roots sampled 2 h after the start of the light period, a moment in which the daily increase of aquaporins may not yet be evident. Nevertheless, in woody root systems, the older suberized root portion can contribute significantly to water uptake and this role could depend upon the suberisation process occurring in this part of the root organ [24]. Proteomic analysis highlighted that only in 101.14 was there found an increase of a long chain acyl-CoA synthetase, an enzyme involved in suberin biosynthesis [63].

### Root growth and hormones

Hormones have a central role in the plant responses to environmental stimuli. Beyond ABA, which plays a substantial role in water stress, other hormones such as auxin, gibberellins (GAs) and jasmonate (JA) are involved in the responses to this abiotic stress ([64] and references therein). Our analysis revealed changes in the abundance of proteins involved in hormone metabolism, which overall suggests a decrease in GAs biosynthesis and an increase in that of JA in both genotypes under stress. These possible changes appear consistent with the reduction in root growth and with the typical responses to stress conditions [65, 66]. Moreover, the proteomic analysis revealed an increase in abundance of an auxin-induced protein PCNT115 isoform 1, which was considerably higher in M4 than in 101.14. This result fits well with the concomitant appearance of an auxin-repressed 12.5 kDa protein and the decrease in an indole-3-acetic acid-amido synthase GH3.1 that occurred only in 101.14. Recently, a central role of auxin-induced protein PCNT115 in the formation of new adventitious roots in chrysanthemum cuttings was proposed [67]. This finding sustains the idea that this protein could play the same role in the root of the grapevine plant.

### Carbohydrate metabolism and plastidial functionality

Water stress deeply affected carbohydrate metabolism: a higher abundance of enzymes involved in the pathways of starch breakdown and sucrose synthesis was observed. This greater use of root storage starch could be a consequence of the reduction of sugar provision from the leaf organ, a result of the fall in net CO_2_ assimilation previously described by Meggio et al. [29]. Nevertheless, these changes at the root level were more evident in M4, even while this genotype maintained photosynthetic activity under more severe WS conditions [29]. Previously, Regier and co-workers found that a crucial characteristic of Poli, a black poplar clone tolerant to WS, was its capability to maintain the photosynthetic rate as well as to improve adequately the usage of carbon skeletons in the root [27]. In agreement with these results, M4 showed a similar behaviour in response to WS.

Nonphotosynthetic plastids are the sites of primary pathways, such as those involved in the synthesis of starch and fatty acids and in nitrogen assimilation [68]. Proteomic analysis suggests a clear reduction in these processes in WS. Some of the plastidial enzymes involved in the production of reducing power (i.e.*,* glucose-6-phosphate 1-dehydrogenase and ferredoxin-NADP reductase) or in plastidial glycolysis (i.e., triosephosphate isomerase and enolase) decreased in abundance in both genotypes. Moreover, some enzymes involved in nitrogen assimilation (i.e., ferredoxin-nitrite reductase and glutamine synthetase in 101.14 and M4, respectively) also decreased in WS.

In WS, a parallel reduction in the abundance of several enzymes involved in fatty acid biosynthesis occurred. Although this effect was observed in both genotypes, the concomitant increase of the glyoxysomal fatty acid beta-oxidation multifunctional protein MFP-a, evident only in 101.14, may suggest that in this genotype lipid catabolism was higher than in M4, possibly due to a different strategy to sustain energetic requirements (i.e., lipid respiration).

Taken together, these data are consistent with a reduction in plastidial functionality that could be directly due to a reduction in abundance of specific enzymes while at the same time there could be an inability to sustain the demand for reducing power by the anabolic processes [68]. According with a shift in the metabolic ways of sustaining the request of NADPH, an increase in cytosolic glucose-6-phosphate dehydrogenase occurred in both genotypes.

### Mitochondrial functionality

Proteomic analysis revealed severe changes in mitochondrial functionality. Indeed, WS induced a fall of the intermediates of the tricarboxylic acid (TCA) cycle in both genotypes. At the same time, a few enzymes of this pathway, such as ATP-citrate synthase and a fumarate hydratase, increased in abundance, while others, such as components of the pyruvate dehydrogenase complex, were adversely affected by WS. This last effect was more evident in 101.14, where many of the components of this enzyme complex decreased under the stress. Conversely, only in M4, other enzymes of the TCA cycle, such as isocitrate dehydrogenase and 2-oxoglutarate dehydrogenase, as well as the anaplerotic enzyme PEP carboxylase, increased in WS, suggesting that this genotype was generally able to maintain a better functionality of the TCA cycle in WS. According to other work [39], this hypothesis also seems to be supported by the increase in mitochondrial formate dehydrogenase observed in M4.

Under stress conditions, a concomitant increase in the levels of several amino acids occurred. This result fits well with the central role of the TCA cycle in providing carbon skeletons for amino acid biosynthesis [69]. Indeed, it could be observed that the higher activation of the TCA cycle hypothesized in M4 is associated with a higher accumulation of specific amino acids, such as valine, threonine, serine, proline, and methionine. Nevertheless, WS negatively affected a larger number of enzymes involved in amino acid metabolism in 101.14 than in M4. In this view, it may be considered that the increase in amino acid levels detected in the stress condition could be, at least partly, a consequence of an increase in protein degradation. This event could be ascribable to general cell damage and/or could represent a specific response evoked by the requirement for alternative substrates for respiration [70, 71].

Although the energy demand is expected to decrease as a consequence of a reduction in root growth and ion uptake, two events frequently affected when WS becomes severe, the maintenance of an adequate mitochondrial respiration is fundamental to sustain the cell functionality ([72] and references therein). Our study reveals that a deficiency in respiratory substrates could occur and, at the same time, only in 101.14 the mitochondrial electron chain (i.e., disappearance of the succinate dehydrogenase [ubiquinone] iron-sulfur subunit 1, mitochondrial) was negatively affected by WS. Overall, the data suggest that, whereas in 101.14 a reduction in the functionality of respiratory machinery emerged, M4 showed a better capability for sustaining the demand for energy.

### Protein metabolism

Changes in both protein catabolism (i.e., increase in abundance of regulatory proteins of the 26S proteasome and of proteases) and synthesis (i.e., changes in abundance of ribosomal proteins and of translation factors) occurred. Nevertheless, the changes in other proteins, such as transport protein SEC24-like, proteins belonging to PRA1 family, coatomer subunits, proteases and other ribosomal proteins, highlighted the occurrence of broad changes in overall protein metabolism. Moreover, only in M4, an increase in basal transcription factor 3 (BFT3), involved in transcription initiation, translational regulation and protein localization and known to be modulated under stress conditions ([73] and references therein), was found. Taken together, the results suggest that in 101.14, protein catabolism prevails, whereas M4 is characterized by a prevalence of protein synthesis and by a greater capability to maintain the vesicle traffic functionality. In this view, a very interesting difference between the two genotypes is the higher number of ribosomal proteins identified and positively affected by WS in M4. This result encourages further studies to deepen knowledge about the changes induced by WS in the cytosolic ribosomal proteome(s) under both the quantitative and the qualitative points of view ([74] and references therein).

### Secondary metabolism and oxidative stress responses

Many differences between the two genotypes concerned proteins involved in secondary metabolism. The main result consisted in an increase of enzymes involved in the synthesis of flavonoid and stilbene compounds, which occurred only in M4. These data are in agreement with the previous transcriptomic study on 101.14 and M4 genotypes grown in the same WS experimental conditions, contributing to reinforce the conclusion that the capability to synthesize larger amounts of antioxidant compounds, such as flavonoids and stilbenes (i.e. resveratrol), enhances the tolerance to WS in M4 [30]. Furthermore, it is interesting to observe that only in M4 was there found an increase of typical ROS scavenging enzymes, such as catalase and glutathione reductase. In other words, the greater tolerance of this genotype appears also to be linked to the ability to activate mechanisms capable of better counteracting the oxidative stress occurring in WS conditions [17, 26, 30, 75].

### Stress-related proteins

Water stress induced changes in several stress-related proteins that were quite different in the two genotypes. M4, when compared to 101.14, showed a larger number of stress-related proteins belonging to Bet v I family, such as MLP proteins that increased in WS. The possible role played by these proteins, in the response to abiotic and biotic stresses is emerging [55, 76–78]. For example, the central role of MLP43 in the modulation of the ABA response to drought conditions has been recently highlighted [55]. Although further work is necessary, it may be hypothesized that the higher tolerance of M4 to WS is also related to the presence of specific MLP-like proteins.

Moreover, the rise of chitinases in the root observed under drought was related to a response useful to reduce the risk of infection in drought-weakened plants. [79, 80]. Our study shows an increase in two chitinases only in the M4 genotype.

Almost all of the stress-related proteins of the germin subfamily decreased in WS in both genotypes. Further work is needed to explain this result, considering that GLPs are a very heterogeneous class whose members show very different characteristics, such as oxalate oxidase activity, superoxide dismutase activity and other unclarified roles involved in the photoperiodic and abiotic stress responses [81, 82].

As previously observed in typical stress conditions [83], an increase in a few heat-shock proteins occurred in both genotypes. The changes observed in 101.14 and in M4 involved different members of this protein family, reaffirming peculiarities in the strategy and/or different abilities of each genotype to respond to WS conditions. Moreover, it is interesting to observe that the three small heat-shock proteins (sHSPs) which appeared or dramatically increased in the 101.14 genotype have a Hsp20/α-crystallin domain, which characterized some sHSPs strongly induced by heat and oxidative stress [84].

## Conclusion

**Fig. 6:**
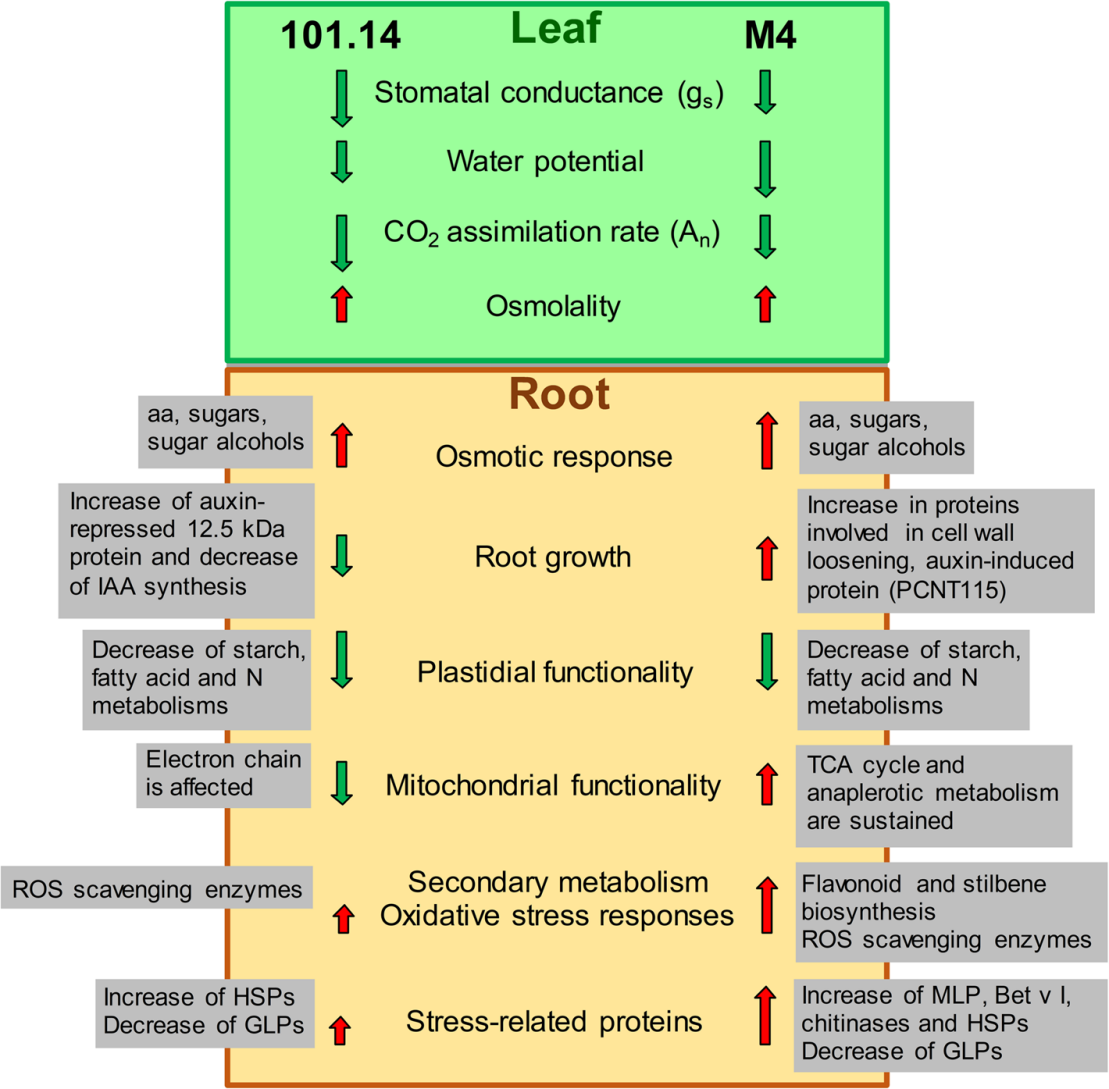
Schematic overview of the responses of 101.14 and M4 induced by WS. The physiological evaluation is reported according to Meggio et al. [29]. Boxes summarize some of the particular responses as well as some of the proteins involved

This study provides new information about the responses to WS in soil growth conditions of the roots of a woody plant, i.e.*,* grapevine. Comparative analysis of two genotypes with different tolerance to this abiotic stress highlighted specific traits of the strategy adopted to counteract it (Fig. 6). The proteomic/metabolomic analyses strengthen the view that a crucial aspect is the capability to activate and to sustain the metabolic pathways involved in the protection of the cell from hazardous events, like a drop in cell turgor, increased oxidative stress and loss of cell structural integrity. Within this framework, the root has to sustain metabolic activities in a condition of reduced carbon skeleton availability, which derives from the reduction in plant photosynthetic performance. In other words, the root must optimize the availability of metabolic energy and sustain as much as possible root functionality and growth. A crucial aspect in the modulation of root responses is the hormonal balance, which controls these physiological/biochemical processes.

An important aspect emerging from this study is that the responses of the root to WS depend on its ability to guarantee mitochondrial functionality, essential for both respiration and anabolic processes. In this view, the activation of alternative pathways capable of sustaining the TCA cycle and the production of reducing power appear to be distinctive responses of the tolerant genotype. Moreover, the observed increases in several stress-related proteins, somewhat different in the two genotypes studied, confirm the multifaceted and very important role of these proteins in the responses to WS.

## Methods

### Root material and water-deficit treatment

Two-year-old grapevines (genus *Vitis*) of rootstocks 101.14 Millardet et de Grasset (*V. riparia* x *V. rupestris*) and M4 [(*V. vinifera* x *V. berlandieri*) x *V. berlandieri* cv. Resseguier no. 1] were grown in pots filled with a sand-peat mixture (7:3 in volume) using experimental conditions previously described by Meggio and co-workers [29].

Plants of each genotype (3 plants for each replicate) used as control (C) were maintained at 80% of soil field capacity while in pots of plants subjected to water stress (WS) the water supply was progressively reduced until down to 30% of field capacity. In order to maintain the established soil water content (SWC), an adequate quantity of water was added twice a day, at 8:00 h and at 18:00 h. After 10 days, starting at 9:00 h (2 h after the start of the light period), plants were sampled immediately after in vivo measurements [29]. Root samples were obtained harvesting the whole root system. The soil was removed from roots by a gentle shaking action. After that, the whole root system was rinsed twice in distilled water, immediately blotted with paper towels, weighed and then frozen in liquid nitrogen and stored at − 80 °C until use.

### Protein extraction

Frozen powdered samples (1 g) of four replicates for each experimental conditions were finely powdered in liquid nitrogen using a pestle and mortar, then to each was added 5% (w/w) of polyvinylpolypyrrolidone, and the total protein fraction was extracted as described by Prinsi et al. [85]. Protein samples were then dissolved in SDS-buffer [150 mM Tris-HCl pH 6.8, 10% (w/w) glycerol, 2% (w/w) Sodium Dodecyl Sulphate (SDS), 2% (v/v) 2-mercaptoethanol] and incubated at 95 °C for 5 min. The sample was centrifuged at 10,000 *g* for 10 min and the supernatant stored at − 80 °C until further use. The protein concentration was determined by the 2-D Quant Kit (GE Healthcare).

### One-Dimensional Gel Electrophoresis (1-DE) and tryptic digestion

The SDS-PAGE (SDS - PolyAcrylamide Gel Electrophoresis) was performed as described by Laemmli [86] in a Hoefer™ SE 600 Ruby Vertical system. Each protein sample, was purified in 10% acrylamide gel (10% T, 2.6% C, 180x160x1 mm). Analytical running was conducted at 200 V at 20 °C until the bromophenol blue line ran off. Proteins were stained using the colloidal Coomassie Brilliant Blue G-250 procedure, as previously described by Neuhoff and co-workers [87]. Electrophoresis was monitored using the Full-Range Rainbow Markers (Mr 12,000–225,000) (GE Healthcare).

The blank portions of the gels as well as the regions above 150 KDa or below 12 KDa were removed from the gel, obtaining a line of about 12 cm for each sample. Then each line was cut in 15 regular slices (8x10x1 mm). In the further analysis each slice was treated as an independent sample. In-gel digestion of the slices was performed according to Prinsi and co-workers [88] with the only refinements consisting in the additionally cutting of the slice into 8 portions and in the volume adjustment, in order to assure that the gel pieces were completely immersed in the treatment solutions. The extracted peptides were finally dissolved in 10 μl of 0.1% (v/v) of formic acid (FA).

### Protein mass spectrometry analysis

All mass spectrometry experiments were conducted on an Agilent 6520 Q-TOF mass spectrometer equipped with an HPLC Chip Cube source driven by a 1200 series nano/capillary LC system (Agilent Technologies). Both systems were controlled by MassHunter Workstation Acquisition (version B.02.01, B2116.20; Agilent Technologies). The chip consisted of a 40-nL trap column and a 75 μm × 150-mm analytical column (Zorbax SB, C18, 300 Å). Peptides were loaded onto the trap column at 4 μL min^− 1^ in 5% (v/v) acetonitrile and 0.1% (v/v) FA. The chip was then switched to separation, and peptides were eluted into the mass spectrometer during a 43-min acetonitrile gradient (from 5 to 50% v/v) in 0.1% (v/v) FA at 0.4 μl min^− 1^. The mass spectrometer ran in positive ion mode and MS scans were acquired over a range from 300 to 3000 mass-to-charge ratio (m/z) at 4 spectra s^− 1^. Precursor ions were selected for auto-MS/MS with a maximum of 4 precursors per cycle and active exclusion set at 2 spectra and released after 0.1 min.

Analysis of MS/MS spectra were performed by Spectrum Mill MS Proteomics Workbench (Rev A.03.03.084; Agilent Technologies). Carbamidomethylation of cysteines was set as fixed modification while variable modification was oxidation of methionines. Trypsin was selected as enzyme for digestion, accepting 2 missed cleavages per peptide. The search was conducted against the subset of *Vitis vinifera* protein sequences (*77,487 entries*) downloaded from the National Center for Biotechnology Information (http://www.ncbi.nlm.nih.gov/). The database was concatenated with the reverse one. The threshold used for peptide identification was Spectrum Mill score ≥ 9, SPI% ≥ 70%, difference between forward and reverse scores ≥2 and error mass shift comprised between ±10 ppm. Peptide quantification was obtained as the Spectrum Intensity (SI) of the precursor (MH^+^). Protein quantification was obtained summing the SI of all identified peptides and normalized as the % respect the sum of all validated proteins in the sample (%(SI), summing all valid peptides in the 15 slices of each lane). Obviously, within each sample some redundant entries were found. If one entry was repeated in two vertically adjacent slices, the two single quantifications were summed to reduce the interference of the cut procedure. In all other cases, the entries were treated as independent form of the same protein (among them, only forms representing at least the 80% of the total protein were considered for quantitative purposes). Only proteins showing at least a two-fold change in their %(SI) (Student’s t-test, *p* < 0.05) were considered subjected to a significant change in abundance.

### MapMan visualization

The proteomic data were visualized in figures reporting schematic metabolism pathways that were produced using the MapMan software [34]. The MapMan software (Version 3.6.0RC1) for local application was downloaded from http://mapman.gabipd.org/ Web Site. Mapping file, kindly provided by Živa Ramšak (see acknowledgement), was prepared starting from *Vitis vinifera* protein sequences present in NCBI and using the BLASTp algorithm to match grapevine proteins with Arabidopsis and potato ones. The matching grape/Arabidopsis or grape/potato were kept only if E-value of the BLASTp match was lower than 10^− 20^. When a grapevine sequence did not have any hits in either TAIR9 or potato ITAG, then it was automatically placed into 35.2 (not assigned/unknown).

### Metabolite analysis

Metabolites were extracted considering the polar fraction derived from 150 mg of frozen powder, according to the protocol by Lisec and coworkers [89] with some modifications, as previously described [90]. The analyses were performed on three replicates through a GC-MS approach using the instrument comprising the gas chromatograph 7890 and the single-quadrupole spectrometer 5975 (Agilent Technologies).

Chromatograms and spectra were evaluated through the software MetaboliteDetector version 2.0.6 beta [91]. After the conversion of Agilent D files to netCDF, the chromatograms were aligned according to the elution of the C8-C40 Alcane Mixture and compound spectra were isolated through deconvolution [peak threshold: 10; minimum peak height: 2, number of bins per scan: 10; deconvolution width (scans): 8.0]. Metabolites were identified matching spectral and retention index (RI) information to a library containing information about the entries of the GMD 20111121VAR5 ALK MSL provided by the Golm Metabolome Database [92], setting the cutoff score to 0.90 and the max RI difference to 15. Identified metabolites were quantified integrating the peak area of the ions normalized by the one of ribitol.

Sucrose level was estimated by colorimetric procedure. Briefly, sucrose was extracted as previously described in Prinsi et al. [88] and then estimated from the difference between total and reducing sugars that were determined according to Nelson method [93].

Student’s t-test (*p*-value < 0.05) was performed through Statistica software v 8.0 (StatSoft Inc., Tulsa) to determine significant differences between means of stressed and well-watered samples.

The results were visualized through the two-way hierarchical clustering methodology using the software PermutMatrix [94, 95]. For this purpose, the data were converted into a binary matrix replacing the values that did not show significant differences by zero. Pearson’s distance and Ward’s algorithm were used for the analysis.

## Additional files

Additional file 1: **Table S1.** Technical parameters concerning peptide validation and protein identification. (PDF 133 kb)

Additional file 2: Quantification data details of all the proteins identified in roots of 101.14 and M4 rootstock genotypes. (XLSX 226 kb)

Additional file 3: Assignment of the functional bin code category of all proteins identified in roots of 101.14 and M4 rootstock genotypes (Table S3A and B) and functional classification of proteins showing significant changes in responses to WS (Table S3C). (XLSX 190 kb)

Additional file 4: Data details of metabolites identified in roots of 101.14 and M4 rootstock genotypes. (XLSX 19 kb)

## Abbreviations

ABA: Abscisic acid
FA: Formic acid
GABA: γ-Aminobutyric acid
GMD: Golm Metabolome Database
GST: Glutathione S-transferases
NCBI: National center for biotechnology information
nLC-nESI-MS/MS: Nanoflow liquid chromatography-mass spectrometry
PAGE: PolyAcrylamide Gel Electrophoresis
ROS: Reactive Oxygen Species
SDS: Sodium dodecyl sulfate
SI: Spectrum Intensity
WS: Water stress
