# Supplementary material for: Root proteomic and metabolic analyses reveal specific responses to drought stress in differently tolerant grapevine rootstocks
Source: BMC Plant Biol. 2018 Jun 20;18:126. doi: 10.1186/s12870-018-1343-0 (PMC6011575; doi:10.1186/s12870-018-1343-0)
Supplement: Supplementary file 1 — Table S1. Technical parameters concerning peptide validation and protein identification. (PDF 133 kb) [file 12870_2018_1343_MOESM1_ESM.pdf]

Table S1. Technical parameters concerning peptide validation and protein identification by nLC-nESI-MS/MS in roots of 101.14 and grapevine rootstocks.

	**101.14**	**M4**
**Peptides**		
Dynamic range of peptide intensity	$1.23x10^{4}$ – $6.89x10^{7}$	$1.33x10^{4}$ – $2.44x10^{7}$
Average peptide intensity	9.14 ± 0.10 ($x10^{5}$)	6.02 ± 0.06 ($x10^{5}$)
Average of MH^{+} error (ppm)	3.21 ± 0.01	3.49 ± 0.02
Average of Scored Peak Intensity	86.79 ± 0.04	82.23 ± 0.04
**Proteins**		
Dynamic range of protein intensity	$2.10x10^{4}$ – $1.96x10^{8}$	$3.74x10^{4}$ – $7.78x10^{7}$
Average range of peptide intensity	3.52 ± 0.10 ($x10^{6}$)	2.27 ± 0.07 ($x10^{6}$)
Protein coverage (%)	15.8 ± 0.3	15.9 ± 0.4
N. unique peptides / protein	4.6 ± 0.1	5.3 ± 0.1
Protein Score (Spectrum Mill)	79.7 ± 1.8	78.8 ± 2.0
Identified unique entries	972	788
